# AI Video Analysis in Parkinson’s Disease: A Systematic Review of the Most Accurate Computer Vision Tools for Diagnosis, Symptom Monitoring, and Therapy Management

**DOI:** 10.3390/s25206373

**Published:** 2025-10-15

**Authors:** Lazzaro di Biase, Pasquale Maria Pecoraro, Francesco Bugamelli

**Affiliations:** 1 Operative Research Unit of Neurology, Fondazione Policlinico Universitario Campus Bio-Medico, Via Alvaro del Portillo 200, 00128 Roma, Italy; 2 Brain Innovations Lab, Università Campus Bio-Medico di Roma, Via Álvaro del Portillo 21, 00128 Rome, Italy; 3 Research Unit of Neurology, Neurophysiology and Neurobiology, Department of Medicine and Surgery, Università Campus Bio-Medico di Roma, Via Alvaro del Portillo 21, 00128 Roma, Italy

**Keywords:** Parkinson’s disease, machine learning, computer vision, quantitative analysis, diagnosis, telemonitoring, therapy management

## Abstract

**Highlights:**

**What are the main findings?**

- Across 45 eligible studies, gait was the most investigated task (followed by bradykinesia), and OpenPose/Custom pipelines were the most commonly used pose-estimation tools.
- Computer Vision pipelines achieved diagnostic discrimination and severity tracking that aligned with clinician ratings, supporting feasibility for both remote and in-clinic assessment.

**What is the implication of the main finding?**

- Computer Vision provides objective, scalable, and non-invasive quantification of PD motor signs to complement routine examination and enable telemonitoring workflows.
- Real-world translation will require standardized video acquisition/processing and external validation across multiple settings and populations.

**Abstract:**

**Background.** Clinical assessment of Parkinson’s disease (PD) is limited by high subjectivity and inter-rater variability. Markerless video analysis, namely Computer Vision (CV), offers objective and scalable characterization of motor signs. We systematically reviewed CV technologies suited for PD diagnosis, symptom monitoring, and treatment management. **Methods.** Following the Preferred Reporting Items for Systematic Reviews and Meta-Analyses guidelines, we searched PubMed for articles published between 1 January 1984 and 9 May 2025. We used the following search strategy: (“Parkinson Disease” [MeSH Terms] OR “parkinson’s disease” OR “parkinson disease”) AND (“computer vision” OR “video analysis” OR “pose estimation” OR “OpenPose” OR “DeepLabCut” OR “OpenFace” OR “YOLO” OR “MediaPipe” OR “markerless motion capture” OR “skeleton tracking”). **Results.** Out of 154 identified studies, 45 met eligibility criteria and were synthesized. Gait was assessed in 42% of studies, followed by bradykinesia items (17.7%). OpenPose and custom CV solutions were each used in 36% of studies, followed by MediaPipe (16%), DeepLabCut (9%), YOLO (4%). Across aims, CV pipelines consistently showed diagnostic discrimination and severity tracking aligned with expert ratings. **Conclusions.** CV non-invasively quantifies PD motor impairment, holding potential for objective diagnosis, longitudinal monitoring, and therapy response. Guidelines for standardized video-recording protocols and software usage are needed for real-world applications.

## 1. Introduction

To date, Parkinson’s disease (PD) diagnosis relies primarily on the identification of cardinal motor symptoms—bradykinesia, rest tremor, and rigidity [1,2,3]. Despite the refinements introduced in the current diagnostic criteria, including the Movement Disorder Society (MDS) criteria, a diagnostic error rate of approximately 20% persists, even in specialized centers [4,5]. Diagnostic accuracy tends to exceed 90% when the clinical diagnosis is performed by a movement disorders expert at the final disease stages [4,6,7,8]. This is largely attributable to the absence of validated objective biomarkers and the intrinsic limitations of clinical observation. Notably, the latest attempt at PD diagnosis is based on a “biological definition”, with two similar but diverging frameworks:

- Neuronal α-synuclein disease (NSD) integrated staging system (NSD-ISS) [9];
- SynNeurGe model [10].

According to these pipelines, the biological definition of PD arises from detecting pathological α-synuclein through seed amplification assay (SAA), while staging and classification combine the following:

- Neurodegeneration: evidence of loss of dopaminergic neurons on neuroimaging;
- Genetic risk.

In routine clinical practice, the assessment of disease severity and progression still relies on semi-quantitative rating scales such as the MDS-Unified Parkinson’s Disease Rating Scale (MDS-UPDRS), which, although widely adopted, suffer from coarse-grained ordinal scoring, low sensitivity to subtle motor changes, and significant inter- and intra-rater variability [11,12]. Indeed, the within-subject reliability of MDS-UPDRS-III has been proven to range between 0.13 and 0.62, confirming a significant amount of error variance [12].

In this context, tremor remains the most common motor manifestation across the spectrum of movement disorders [13]. A variety of clinical and neurophysiological classifications have been proposed to distinguish parkinsonian tremor from essential tremor and dystonic tremor [13,14,15,16,17,18,19,20,21]. However, overlap in presentation, variability in phenomenology, and patient-specific fluctuations often limit the diagnostic value of clinical observation alone [22,23]. These limitations become even more pronounced in the context of home monitoring or during fluctuating responses to dopaminergic therapy, where symptoms such as bradykinesia, freezing of gait (FoG), or dyskinesias may go unnoticed during short in-clinic visits [24,25].

Several wearable technologies have been developed to provide quantitative and continuous monitoring of PD motor symptoms [26]. These include inertial measurement units (IMUs), surface electromyography (EMG), and smart textiles [27,28]. Such devices have shown potential in capturing tremor [14,15,19,29], bradykinesia [30], rigidity [31,32,33], and postural instability [34,35,36,37,38]. Additionally, multimodal integration with electroencephalography (EEG) [39] may enhance phenotypic classification and therapeutic response. However, these solutions are often cumbersome, require technical expertise, and may interfere with natural movement, limiting their widespread clinical application [40]. Adaptive and closed-loop-based strategies have been addressed for the management of motor complications in PD [41] to overcome conventional continuous pharmacological/electrical stimulation paradigms [42,43,44,45,46,47]. These innovative methods dynamically adjust therapeutic regimens in real-time, leveraging biomarker-driven feedback to ensure optimal motor performance is maintained throughout the entire day. A key aspect of this approach is identifying robust biomarkers that reliably correlate with and are sensitive to variations in motor states.

In this context, the growing availability of low-cost, high-resolution video acquisition tools has opened new avenues for remote and unobtrusive assessment. On this purpose, artificial intelligence (AI) [48,49], and particularly machine learning (ML), has been employed to extract clinically relevant features from human motion not only in PD, but also in the stroke field [50,51,52]. These models can characterize the severity of motor symptoms [53], identify subtypes, and even optimize pharmacologic regimens by detecting medication-related motor fluctuations [54,55]. For example, Pfister and colleagues [56] applied deep learning to wrist-worn IMU data to monitor tremor in free-living conditions, achieving high classification performance. Nevertheless, even wearable-based AI solutions face limitations in compliance and long-term usability [28].

The urgency for scalable, objective, and ecologically valid tools has thus directed increasing attention to video-based assessment methods [57]. These tools aim to preserve the naturalistic context of movement while enabling automated and reproducible motor symptom quantification [58]. Among these, markerless automated video analysis, namely Computer Vision (CV), represents the most promising solution.

### 1.1. Computer Vision

**Figure 1:**
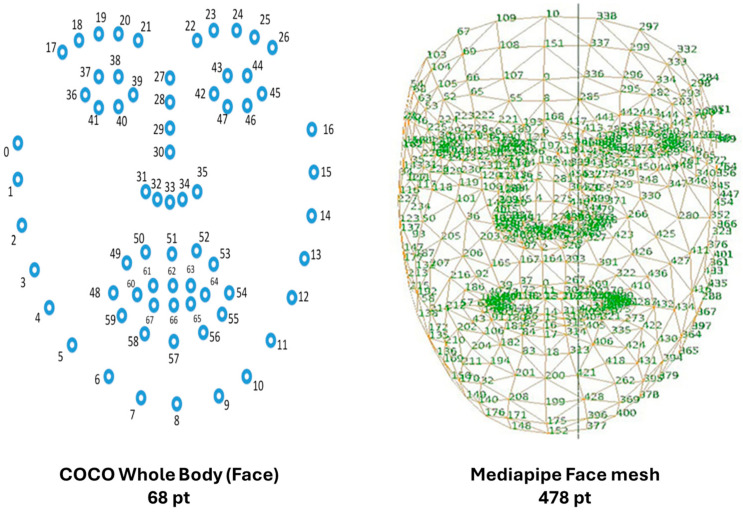
Face landmark models for 2D and dense 3D facial pose estimation. For the COCO Whole Body Face model, the numbering ranges from 24 to 91. In the current image, it has been simplified from 0 to 67.

CV methods applied to PD rely on a multi-step pipeline that begins with human pose estimation and proceeds through feature extraction, data preprocessing, and ML-based classification or regression. This approach enables the transformation of raw video frames into clinically meaningful predictions, offering an automated, markerless, and scalable solution for motor assessment. The foundation of CV-based movement analysis lies in human pose estimation (HPE), which detects anatomical keypoints—such as wrists, elbows, shoulders, and fingertips—from each frame of a video. Modern HPE models are based on deep convolutional neural networks (CNNs), which learn spatial filters capable of identifying patterns associated with body parts. The output of HPE is typically a time series of 2D or 3D coordinates (X, Y, Z) for each keypoint over time, forming the skeleton trajectory of the subject. Facial analysis relies on a 68-point 2D landmark layout and, when available, a dense 3D mesh to measure perioral and periocular motion, symmetry, and expression dynamics relevant to hypomimia (Figure 1).

**Figure 2:**
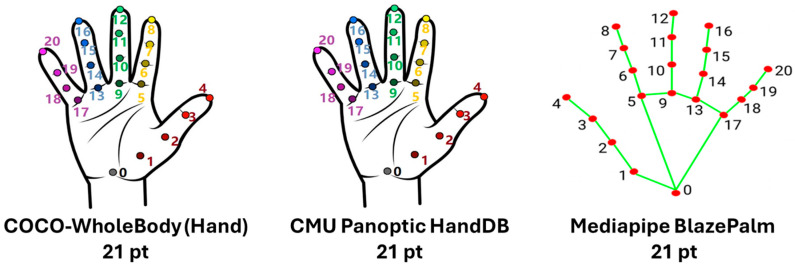
Hand landmark models.

Hand pose is represented with a canonical 21-keypoint template (wrist plus MCP, PIP, DIP, and fingertip landmarks), enabling precise assessment of anatomical reference points (Figure 2).

**Figure 3:**
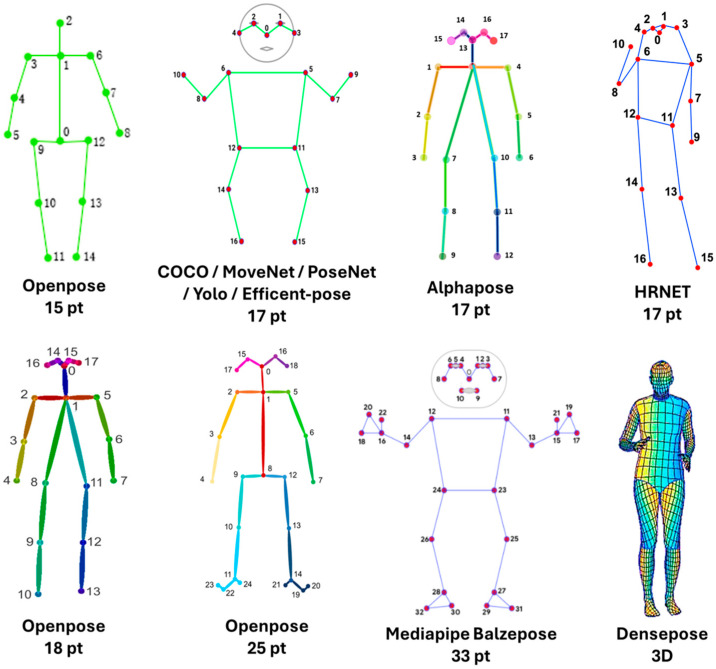
Full-body landmark models.

Two-dimensional skeletal models with 17–33 keypoints covering trunk and limb joints quantify posture, gait, and upper-limb kinematics for whole-body pose estimation (Figure 3).

These pose sequences are then passed to the next stage for feature extraction. The extracted keypoints undergo quantitative processing to derive kinematic features. In some studies, handcrafted features are directly fed into ML classifiers. In others, the entire pose time series is input to deep sequence models (e.g., LSTM, GRU, or Transformers) capable of learning latent temporal representations without explicit feature engineering. The goal of ML is to map the kinematic features to diagnostic or severity-related labels. This is accomplished through supervised learning techniques, where the model is trained on annotated datasets with known ground truth (e.g., MDS-UPDRS scores, diagnosis labels, or symptom severity).

CV can support both telemedicine and in-clinic workflows in PD. During video consultations, CV algorithms applied to standard webcam streams can extract kinematic features (e.g., amplitude, speed, rhythm, sequence effect) from routine MDS-UPDRS tasks in near–real time, enabling objective scoring alongside clinical observation. In face-to-face visits, the same pipelines can be run on videos captured with commodity cameras to deliver quantitative kinematic analysis offline, facilitating standardized documentation, longitudinal tracking, and response-to-therapy assessment. These dual-use deployments require minimal calibration and integrate with existing clinical practice, improving reproducibility while imposing negligible additional burden on patients or clinicians.

### 1.2. Human Pose Estimation: Datasets and Frameworks

The progress of pose estimation has been inseparable from the release of large annotated datasets. The Microsoft COCO dataset [59] remains the dominant benchmark, offering over 200,000 images with 250,000 annotated person instances and 17 2D keypoints, enabling robust multi-person training in unconstrained environments. The MPII Human Pose dataset [60] contributed around 25,000 images with 40,000 annotated poses and 14–16 keypoints, particularly focused on human activities. Human3.6M [61] provided 3.6 million annotated frames with accurate 3D ground truth from motion capture systems in controlled laboratory settings, crucial for the development of 3D estimators. Another landmark is the CMU Panoptic Studio dataset [62], containing multi-camera recordings with synchronized 2D and 3D annotations across 100+ views, specifically enabling OpenPose to expand to whole-body pose with body, hand, foot, and facial landmarks. More targeted datasets enriched the ecosystem: AI Challenger [63] expanded scale with 300,000 images; CrowdPose [64] tackled occlusion and dense crowds; PoseTrack [65] introduced pose tracking across frames; and DensePose-COCO [66] extended sparse COCO annotations to dense pixel-to-surface mappings. COCO WholeBody [67] extends the COCO Keypoints benchmark to 133 anatomical landmarks covering the full body, feet, face, and hands. By providing consistent whole-body annotations, COCO WholeBody enables training and benchmarking of whole-body pose estimators, improving the detection of distal segments and fine-motor patterns. This is particularly relevant for clinical video analysis in PD, where accurate hand and facial landmarking supports the quantification of finger-tapping bradykinesia and hypomimia. Together, these resources established the backbone for pose estimation benchmarks, spanning from controlled lab-based 3D data to real-world, multi-person and occluded scenarios (Table 1).

Frameworks have evolved by leveraging these datasets and progressively expanding the number of landmarks and the dimensionality of pose estimation. OpenPose [68], developed at CMU and trained on COCO, MPII, and the CMU Panoptic Studio dataset, was the first to offer real-time multi-person estimation. It introduced multiple configurations: 15- or 18-keypoint body models, extended to a 25-keypoint whole-body version with six foot keypoints, as well as 21 hand keypoints and 70 facial keypoints. OpenPose works natively in 2D but supports 3D extensions via multi-view setups. PoseNet [69] enables real-time 2D human pose estimation in the browser and on mobile via TensorFlow.js, targeting interactive experiences, fitness tracking, motion-based games, and telemedicine. AlphaPose [70], trained on COCO and MPII, adopted a top-down approach to improve localization in multi-person scenarios, providing 17 keypoints in 2D. HRNet [71], introduced by Microsoft, emphasized high-resolution representations, maintaining 17 body keypoints with state-of-the-art accuracy. MediaPipe, and its extension BlazePose [72,73], offered a lightweight solution optimized for mobile, supporting 33 body landmarks in 3D (x,y,z), combined with the GHU/GHUM generative 3D human shape model [74] to estimate full 3D body pose from single images or video. The pipeline also detects 21 landmarks per hand and up to 478 3D facial landmarks, providing one of the densest anatomical estimators available in real time. YOLO-Pose [75] extended the YOLO object detection family with 17 body keypoints in 2D, with experimental 3D variants. DeepLabCut [76] focused on user-defined landmarks for animal and human research, adaptable for 2D and 3D via multi-camera input. EfficientPose [77] optimized efficiency and speed, supporting 17 COCO body keypoints in 2D. DensePose [66] departed from sparse skeletons, mapping every body pixel to a 3D surface model. MoveNet [78] is a lightweight, real-time 2D model released in two variants—Lightning (low latency) and Thunder (higher accuracy)—and is widely used for mobile/web applications such as interactive fitness and gesture recognition. Collectively, these frameworks illustrate the shift from sparse 2D keypoint detection to richer full-body 3D estimators, with growing emphasis on either precision (HRNet, AlphaPose) or lightweight real-time applications (MediaPipe/BlazePose, EfficientPose) (Table 2).

Beyond movement disorder-specific surveys, broader reviews on markerless motion capture (MMC) and deep-learning video analysis frame the methodological context for clinical adoption. Lam and colleagues [79] systematically reviewed 65 studies applying MMC as a clinical measurement tool and found that MMC is used mainly to identify clinical signs or group differences vs. controls, with PD as the most frequently assessed population; Microsoft Kinect emerged as the most used system, with a recent shift toward smartphone video, while the overall clinical use remains preliminary and demands user-friendly, clinician-interpretable platforms. Moreover, the systematic literature review by Sharma and colleagues [80] synthesized 93 peer-reviewed studies (2011–2020), documenting a sharp acceleration from 2017–2020 driven by CNN/LSTM architectures (e.g., AlexNet/ResNet/LSTM). Hybrid CNN + LSTM models improved spatiotemporal learning, yet progress is constrained by limited public datasets, annotation quality, and compute requirements, which are pivotal to clinical translation. Finally, multi-sensor datasets integrating RGB-D, inertial units (IMU), and mmWave radar specifically for rehabilitation tasks underscore a trend toward multimodal pose estimation that can balance privacy, cost, and accuracy in home or clinic settings. Benchmarks show that sensor fusion outperforms single modalities (all-three modalities best), and that mmWave—while slightly less accurate than RGB/IMU—offers privacy and low-power advantages attractive for home or clinic use [81].

Moreover, comparative studies quantifying agreement between CV-based pipelines and reference technologies showed encouraging—but task- and joint-dependent—accuracy. In overground gait, OpenPose (2D) achieved mean absolute errors of ~0.01–0.02 s for temporal parameters (step/stance/swing/double support), ~0.018–0.049 m for step length, and sagittal hip/knee/ankle angle errors of ~4.0°, 5.6°, and 7.4° versus 3D motion capture, with performance improving when parameters are averaged within participants [82]. A recent head-to-head analysis against an optoelectronic system showed that IMUs generally outperformed vision systems in RMSE and range-of-motion error (εROM), yet modern pose estimators (RTMPose, MediaPipe) approached IMU performance for hip/knee angles (minimum absolute errors ~3.1–4.8°), while ankle angles remained unreliable with vision alone [83]. Together, these studies delineate where CV methods already meet clinically useful precision and where integration with wearables or multimodal sensing may be required.

To our knowledge, no clinically focused, state-of-the-art overview of CV application for PD has been published. Prior reports focused on the global realm of movement disorders [58] or adopted a predominantly bioengineering perspective [84]. To address this gap, we conducted a systematic review that synthesizes the current landscape of CV in PD, organized by type of core symptom analyzed and diagnostic, telemonitoring and therapeutic response paradigms. We critically appraise methodological strengths and limitations, delineate the translational potential of CV for diagnosis and longitudinal monitoring, and provide practical recommendations for routine clinical adoption of markerless video analysis in PD.

## 2. Materials and Methods

### 2.1. Scope and Target Domain

This review targets clinical neurology in movement disorders, with a focus on human, patient-facing applications of CV technology suited for diagnosis and symptom monitoring in PD. This work included studies that: (i) have analyzed videos acquired in clinical or home settings; (ii) have estimated motor features relevant to movement disorder assessment; and (iii) have reported clinically interpretable performance metrics and/or validation against reference systems. Works primarily focused on engineering benchmarks without clinical endpoints, wearable-only studies without a video-CV component, marker-based motion-capture studies (unless used solely as a reference for validating CV), and animal studies were excluded. Therapy management (motor fluctuations, levodopa-induced dyskinesias, therapy response) were summarized separately and not pooled with diagnosis/monitoring accuracy aggregates.

The intended audience is clinicians and translational researchers seeking evidence on the state of the art about feasibility, accuracy, and validation pathways for in-clinic and home-based video-CV assessment.

### 2.2. Search Strategy

This systematic review follows the Preferred Reporting Items for Systematic Reviews and Meta-Analyses (PRISMA) guidelines. We searched PubMed for articles published between 1 January 1984 and 9 May 2025, focusing on English-language articles meeting inclusion criteria. The search string was: (“Parkinson Disease” [MeSH Terms] OR “parkinson’s disease” OR “parkinson disease”) AND (“computer vision” OR “video analysis” OR “pose estimation” OR “OpenPose” OR “DeepLabCut” OR “OpenFace” OR “YOLO” OR “MediaPipe” OR “markerless motion capture” OR “skeleton tracking”).

### 2.3. Eligibility Criteria

A title/abstract screening process was followed by a full-text screening. After screening all studies, all raters merged their findings. No automation tool was used for the process. The eligibility criteria were designed according to the PICO (Population, Intervention, Comparison, Outcome) framework. The following PICO questions were investigated:

1. In studies on PD, what is the performance of markerless CV–based technologies compared to traditional clinical methods for disease diagnosis?
2. In studies on PD, what is the performance of markerless CV–based technologies compared to traditional clinical methods for motor symptom monitoring and severity assessment?

To address these questions, eligibility criteria were defined based on the following parameters:

- Population (P): Patients with PD, diagnosed according to standard clinical criteria, including both early- and late-stage individuals, as well as subgroups with varying motor symptom profiles;
- Intervention (I): Application of markerless CV technologies for motor assessment, including pose estimation algorithms, video-based tracking, and ML-driven motion analysis for the evaluation of motor symptoms;
- Comparison (C): Comparison with traditional methods of clinical assessment, expert rater evaluation, or alternative quantitative tools such as wearable sensors or marker-based motion capture systems;
- Outcome (O): Objective measures of diagnostic or assessment performance, including but not limited to classification accuracy, sensitivity, specificity, correlation with clinical scores, Area Under the Receiver Operating Characteristic Curve (AUROC), regression coefficients, or treatment response quantification.

Original research articles published in English were included. Review articles, conference abstracts (unless full text was provided and met criteria), letters to the editor, and studies not involving human subjects were excluded.

### 2.4. Data Extraction and Synthesis

Key information extracted from each included article comprised:

- Study characteristics: Participant demographics, sample size, and clinical settings;
- Motor tasks assessed: Specific movements or tests;
- CV methodologies: Type of camera used, specific pose estimation algorithms or software employed, and ML models utilized;
- Performance metrics: Reported accuracy, sensitivity, specificity, AUROC, and correlation with established clinical scales.

The extracted data were systematically synthesized by grouping the included studies according to their primary objective. Specifically, each study was categorized into one of three overarching aims:

(1) Diagnosis, if the study focused on distinguishing individuals with PD from healthy controls or other movement disorders based on a single assessment;
(2) Symptom monitoring, if the study involved longitudinal assessments across multiple time points or visits, typically conducted in remote or real-world settings;
(3) Therapy Management, if the aim was to differentiate motor states (e.g., ON vs. OFF vs. dyskinesia) or to assess the effects of specific treatments.

### 2.5. Quality Assessment

The quality of the included studies was rigorously assessed using the Quality Assessment of Diagnostic Accuracy Studies (QUADAS-2) tool. This tool evaluated the risk of bias and applicability across four domains: patient selection, index test, reference standard, and the flow and timing of testing. All 45 included articles underwent a quality check to examine risk of bias and concerns regarding applicability to the review aim and PICO questions.

### 2.6. Reporting Framework and Presentation of Results

Results were synthesized according to a clinician-oriented perspective and application scenarios. In particular, studies were summarized per clinical tasks investigated. Motor tasks were grouped into the following:

- Bradykinesia
- Tremor
- Rigidity
- Axial symptoms
  ○ Facial expressions
  ○ Gait, posture, and balance
- MDS-UPDRS-III total score

Within each of these categories, the findings were further organized based on the specific clinical purpose:

- Diagnosis
- Symptom monitoring

Finally, therapeutic management was classified into the following:

- Motor fluctuations
- Levodopa-induced dyskinesias (LIDs)
- Therapy response

## 3. Results

### 3.1. Study Selection

**Figure 4:**
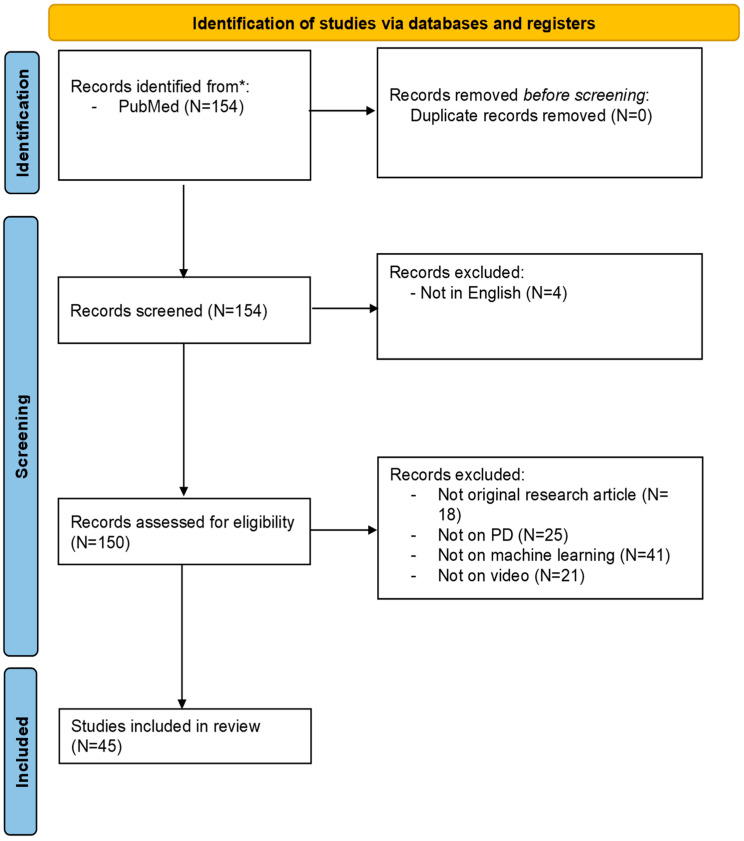
PRISMA flow diagram of literature search and selection process. * = name of searched database.

A total of 154 records were identified through the PubMed database using the predefined search strategy. After title and abstract screening, 103 records were excluded for the following reasons: not original research articles (N = 18), non-English language (N = 4), not focused on PD (N = 25), not related to CV technology (N = 41), and not involving video-based analysis (N = 21). Following this screening process, 45 studies met the eligibility criteria and were included in the final review. The selection process is visually represented in the PRISMA flowchart (Figure 4).

The 45 included studies collectively highlighted the growing utility and advancements of CV in assessing PD motor symptoms. A diverse range of methodologies and applications were observed across various motor tasks. We present the findings following the prespecified scheme outlined in paragraph 2.4: by motor task, and within each motor task, by clinical aim (diagnosis and symptom monitoring).

Included studies were synthesized in tables as follows: aim, number of PD patients and (if present) controls, recording device, pose estimation model, performance, and eventual technology used for validation.

**Figure 5:**
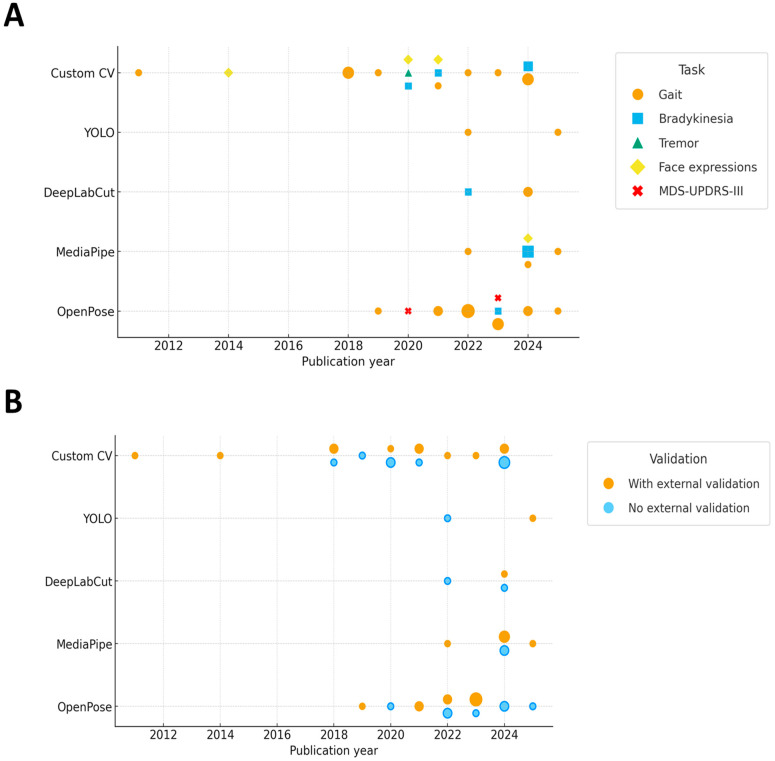
Timeline of methodological iterations in studies on CV for PD over 2011–2025. (**A**) Task timeline by pose estimation model. (**B**) External validation timeline. When multiple studies share the same year–model–category, they are collapsed into a single symbol, whose area is proportional to the number of studies for the year.

Moreover, to graphically contextualize methodological evolution and temporal shifts in models and technology validation across the included studies, we plotted a timeline summarizing, by publication year, the pose estimation model used and the primary clinical task (Figure 5A), and whether external validation against a reference technology was performed (Figure 5B).

The timeline shows that research has been dominated by gait tasks, with fewer studies on bradykinesia, tremor, and facial expressions. Early work relied mainly on OpenPose, followed by diversification toward MediaPipe, DeepLabCut, YOLO, and persistent Custom CV solutions after 2020. External validation against reference systems has increased over time but remains inconsistent, with a substantial fraction of recent studies still lacking it. Overall, the field is expanding in model choice and application scope, yet methodological validation has not uniformly kept pace.

### 3.2. Bradykinesia

**Figure 6:**
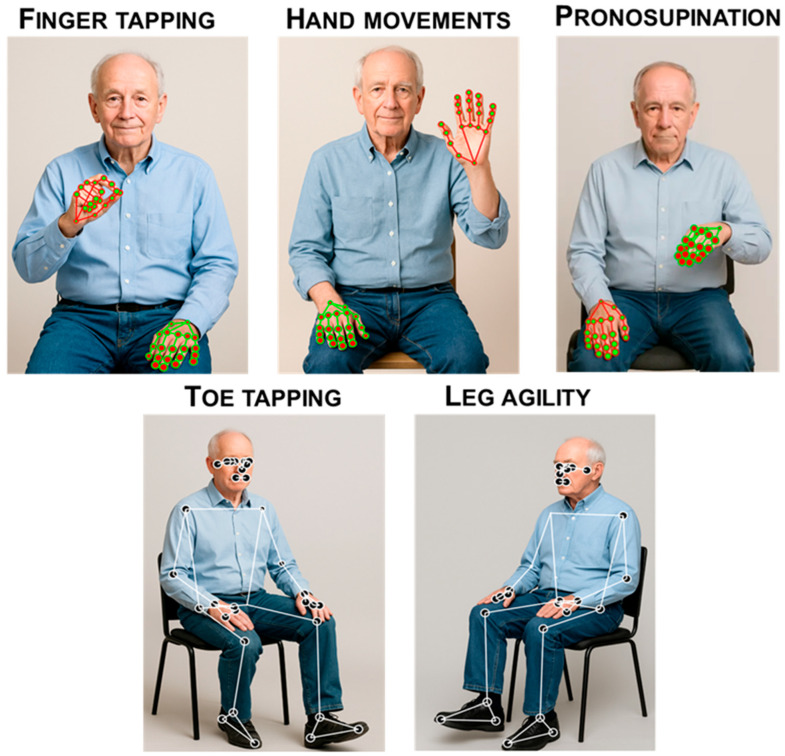
Example CV overlays used to quantify bradykinesia tasks from videos. (**Top Row**) finger tapping, hand movements, and pronosupination task. (**Bottom Row**) Toe tapping and leg agility task. For privacy reasons, the subject photo has been generated with AI (DALL·E 3 model, OpenAI, San Francisco, CA, USA), and the landmarks have been subsequently computed with MediaPipe (MediaPipe, Google, 2023, Version 0.10.26, 10 July 2025).

Bradykinesia is the slowness of voluntary movement, defined as slowness accompanied by a progressive reduction in movement amplitude or speed—or progressive hesitations/halts—during repetitive movements [2]. Motor tasks of MDS-UPDRS-III assessing bradykinesia encompass finger tapping, hand opening/closing, pronosupination of hands, toe tapping, and leg agility (MDS-UPDRS-III items 3.4 to 3.8). CV pipelines can quantify bradykinesia by converting RGB video into frame-wise skeletal landmarks and then deriving kinematic time series. Speed is captured from linear/angular trajectories as instantaneous, mean, and peak velocity, dominant tapping frequency, and movement time per cycle. Rhythm variability is quantified from cycle-to-cycle timing and regularity—including coefficient of variation of inter-tap intervals, short-term variability, spectral bandwidth around the fundamental, entropy/irregularity indices, and autocorrelation decay. Amplitude is computed as peak-to-peak excursion or aperture angle (thumb–index), range of motion, and their dispersion across cycles. The sequence effect is modeled as the progressive decrement across repetitions using the slope of amplitude/velocity vs. cycle index, exponential decay constants, or cumulative-sum change-point metrics, optionally normalized for limb length and camera geometry (Figure 6). These features are aggregated over trials and mapped to severity (MDS-UPDRS-III items) with rule-based thresholds or supervised models (SVMs, gradient boosting, RNN/Transformer encoders), yielding objective, rater-independent estimates of bradykinesia [58,85].

#### 3.2.1. Diagnosis

A deep learning neural network applied directly to videos demonstrated an accuracy of 0.69 and an AUROC of 0.76 for discriminating PD from HC through finger-tapping analysis [86]. Another approach utilizing spatiotemporal 3D hand pose estimation yielded an 81.2% classification accuracy for finger tapping [87].

An earlier study, despite a limited sample size of 13 patients with advanced PD and 6 HC, reported a 95% accuracy using a SVM to discriminate between patients and controls based on tapping features [88].

For early-stage PD, integrating kinematic analyses of left-side, right-side, and bilateral symmetry movements from finger-tapping, hand movement, and leg agility videos resulted in an 86% detection accuracy [89] (Table 3).

#### 3.2.2. Symptom Monitoring

Guarin and colleagues [90] analyzed 180 finger-tapping videos (66 PD, 24 HC) using MediaPipe to extract kinematics from the thumb–index angle. A stage-aware tiered binary classifier outperformed multiclass/ordinal baselines (HC vs. PD: AUC 0.97, F1 0.91; max AUC 0.97). Amplitude and speed declined linearly with severity, whereas variability metrics were nonlinear and best separated higher severities.

Williams and colleagues [91] analyzed smartphone finger-tapping videos (n = 70; 40 PD hands, 30 controls), extracting optical-flow features (frequency, amplitude, variability). An RBF-SVM best classified bradykinesia with 80% accuracy, outperforming Naïve Bayes and logistic regression (Table 3).

**Table 3:** Summary of included studies focusing on bradykinesia assessment classified according to PICO questions per PD patients and controls enrolled, pose estimation software, recording device, performance, and technology used for validation.

Aim	Ref.	Patients	Controls	Device	Pose Estimation	Performance	Technology Comparison
Diagnosis	[86]	40	37	Smartphone (1920 × 1080 px, 60 fps)	Victor Dibia Handtracking, preprocessing in OpenCV	Test accuracy 0.69, precision 0.73, recall 0.76, AUROC 0.76	NA
	[87]	48	11	Intel RealSense SR300 depth camera (640 × 480, ~30 fps, Intel Corporation Santa Clara, CA, USA)	YOLOv3	5-fold CV accuracy 81.2% for 0–4 severity classification	NA
	[88]	13	6	Single fixed camera video (10 s, 25 fps, 352 × 288)	OpenCV Haar face detector + motion-template gradient	Accuracy classification 88% (10-fold CV); PD vs. HC 95%; Head-to-head vs. marker-based: mean peak difference ≈ 3.31 px	Marker-based (HSV) system
	[89]	31	49	Standard clinic video camera	DeepLabCut	AUC 0.968; Sensitivity 91%; Specificity 97%	NA
	[92]	82	61	Video camera consumer Sony HDR-CX470 (30 fps, 1280 × 720, Sony Corporation, Tokyo, Japan)	OpenPose	AUC 0.91, Accuracy 0.86, Sensitivity 0.94, Specificity 0.75. After propensity matching: AUC 0.85; Accuracy 0.83, Sensitivity 0.88, Specificity 0.78	NA
	[93]	31	26	Standard camera (1920 × 1080, 30/60 fps).	MediaPipe	Accuracy: − Finger Tap 79% − Hand Movement 75% − Leg Agility 79% − Combined tasks 86%	NA
Symptom monitoring	[90]	66	24	Standard RGB camera on tripod at 30 fps	MediaPipe	HC vs. PD: AUC-PR 0.97, F1 = 0.91 (accuracy: HC 85%/PD 88%)	NA
	[91]	20	15	Smartphone (iPhone SE, 60 fps, 1080p, Apple Inc, Cupertino, CA, USA)	Hand detector CNN (MobileNetV2 SSD, TensorFlow) + GrabCut	UPDRS-FT > 1 (SVM-R) accuracy 0.80, sensitivity 0.86, specificity 0.74 (LOO-CV) PD diagnosis (NB) accuracy 0.64 (LOO-CV)	NA

Abbreviations: PD = Parkinson’s disease; HC = Healthy controls; NA = not assessed; fps = frames per second; px = pixels; AUC = Area Under the Curve; ROC = Receiver Operating Characteristic; MDS-UPDRS = Movement Disorder Society Unified Parkinson’s Disease Rating Scale; SVM = Support Vector Machine; NB = Naïve Bayes; CNN = Convolutional Neural Network; LOO = Leave-one-out; CV = cross validation.

### 3.3. Tremor

**Figure 7:**
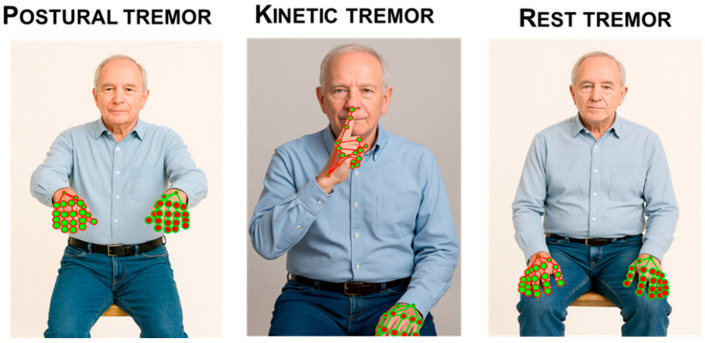
CV overlays for tremor assessment from video. Three task conditions are illustrated with hand-landmark detection (MediaPipe dots/segments): postural tremor, kinetic tremor, and rest tremor. For privacy reasons, the subject photo has been generated with AI (DALL·E 3 model, OpenAI, San Francisco, CA, USA), and the landmarks have been subsequently computed with MediaPipe (MediaPipe, Google, 2023, Version 0.10.26, 10 July 2025).

Tremor is a rhythmic oscillatory movement around an axis [13]. Motor tasks of MDS-UPDRS-III to evaluate tremor encompass postural, kinetic, and rest components (MDS-UPDRS-III items 3.15 to 3.18). CV quantifies tremor by converting ordinary RGB video into motion signals from the hands, fingers, jaw, or legs, then extracting frequency and amplitude features. After stabilizing the clip and isolating the region of interest (e.g., via pose/hand landmarks), frame-to-frame motion is derived with keypoint trajectories or optical flow; subtle oscillations can be enhanced with Eulerian Video Magnification (EVM) when needed. The resulting displacement/velocity traces are band-pass filtered (typical PD tremor 3–7 Hz), and the dominant frequency is estimated from the power spectral density or autocorrelation peak, with metrics such as spectral peak, bandwidth, and temporal stability. Amplitude is summarized as peak-to-peak or root-mean-square (RMS) displacement (in pixels or calibrated units), or as tremor power within the target band. These features can be computed unilaterally or bilaterally to assess asymmetry and rest vs. postural conditions, yielding objective, rater-independent measures of tremor severity from contactless video [58,85] (Figure 7).

#### 3.3.1. Diagnosis

In a blinded study of 48 apparently atremulous hands (26 from 17 PD participants; 22 from 11 controls) recorded on a smartphone at 60 fps, EVM tuned to 3–7 Hz (×20 gain) and rated by three movement disorder neurologists significantly increased correct PD vs. control classification (OR 2.67, 95% CI 1.39–5.17; *p* < 0.003). While magnification also induced apparent tremor in some control hands, the effect was stronger in PD, consistent with visualization of subclinical pathological tremor [94] (Table 4).

#### 3.3.2. Symptom Monitoring

For tremor assessment, a Global Temporal-difference Shift Network (GTSN) model, enhanced by EVM, has shown high accuracy (90.6% for rest tremors in hands) in estimating MDS-UPDRS scores from tremor videos, indicating utility for continuous symptom tracking [95]. Indeed, a smartphone-based method also demonstrated excellent agreement with accelerometry for tremor frequency, with 97% of video measurements within 0.5 Hz of the clinical gold standard [96] (Table 4).

**Table 4:** Summary of included studies focusing on tremor assessment classified according to PICO questions per PD patients and controls enrolled, pose estimation software, recording device performance, and technology used for validation.

Aim	Ref.	Patients	Controls	Device	Pose Estimation	Performance	Technology Comparison
Diagnosis	[94]	13	11	Smartphone (1080p, 60 fps)	EVM	OR 2.67 (95% CI 1.39–5.17; *p* < 0.003)	NA
Symptom monitoring	[96]	15 (9 PD, 5 ET, 1 FT)	-	Smartphone (1080p@60 fps) + hand accelerometer (3.84 kHz)	Optical flow	MAE: 0.10 Hz; 95% LoA −0.38 to +0.35 Hz; 97% within ±0.5 Hz; Bland–Altman bias −0.01 Hz	Accelerometer
	[95]	130	-	Single Sony camera (1920 × 1280, 30 fps, Sony Corporation, Tokyo, Japan)	OpenPose	Accuracy: − Rest hand 90.6% − Leg 85.9% − Jaw 89.0% − Postural hand 84.9%	NA

Abbreviations: PD = Parkinson’s disease; HC = Healthy controls; NA = not assessed; fps = frames per second; OR = Odds Ratio; CI = Confidence Interval; EVM = Eulerian Video Magnification; FT = Functional tremor; MAE = Mean absolute error; LoA = limit of agreement; ICC = Intraclass coefficient.

### 3.4. Rigidity

**Figure 8:**
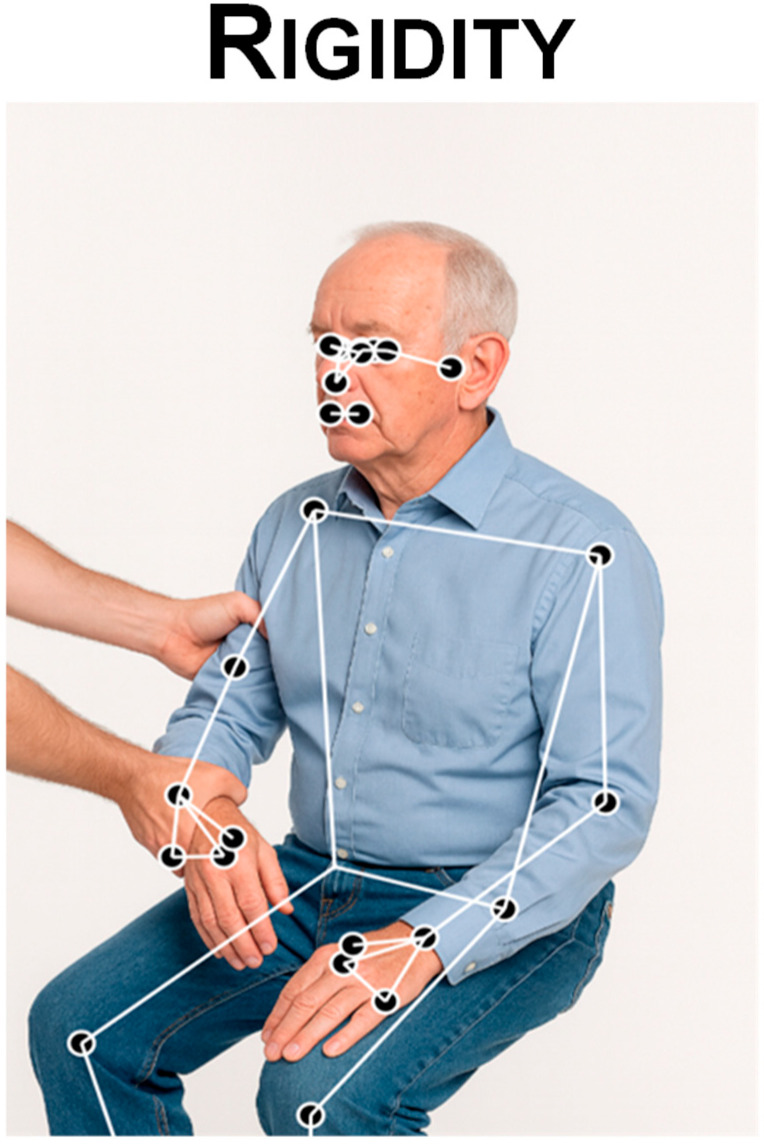
CV overlays for rigidity assessment during clinician-imposed passive movement. For privacy reasons, the subject photo has been generated with AI (DALL·E 3 model, OpenAI, San Francisco, CA, USA), and the landmarks have been subsequently computed with MediaPipe (MediaPipe, Google, 2023, Version 0.10.26, 10 July 2025).

Parkinsonian rigidity is a velocity-independent increase in muscle tone producing “lead-pipe” resistance to passive movement (often with superimposed cogwheeling) [2,97]. According to the MDS rating score system [2], rigidity is specifically judged during slow, passive joint movement with the patient relaxed and should be evaluated separately for the neck and the four limbs individually (MDS-UPDRS-III item 3.3). CV can assess rigidity by quantifying the smoothness of slow joint excursions recorded on video—either during clinician-imposed passive movements or standardized active tasks. After extracting 2D/3D pose trajectories (e.g., wrist–elbow), velocity traces are derived and smoothness metrics are computed, such as spectral arc length (SPARC) or jerk-based indices [97]. These measures mirror inertial-sensor approaches in which reduced smoothness and increased segmentation (“cogwheeling”) track rigidity and ON/OFF state changes; indeed, SPARC from gyroscope data has been shown to discriminate motor states in PD and to relate to rigidity-related kinematic degradation. In video, the same constructs apply to keypoint trajectories. Eulerian Video Magnification (EVM) can amplify subtle stick–slip motion and micro-oscillations of the limb during passive manipulation, improving sensitivity before computing smoothness and low-frequency power features. Overall, CV thus offers a contactless proxy for rigidity by transforming pose-derived speed profiles into objective smoothness-based biomarkers [58,85] (Figure 8).

### 3.5. Axial Symptoms

#### 3.5.1. Facial Expressions

**Figure 9:**
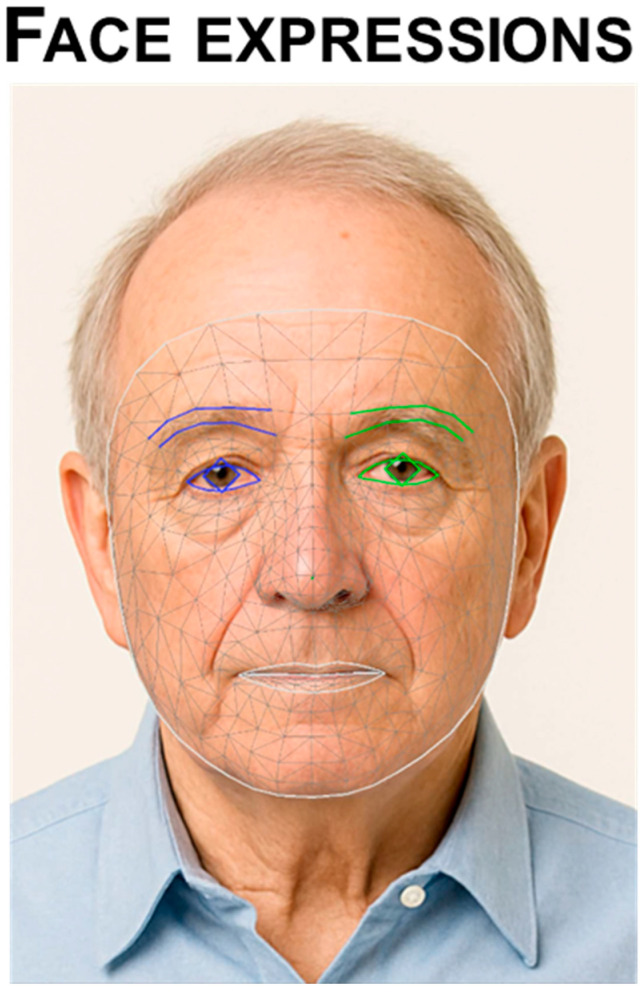
Frontal facial landmarking for CV analysis of facial motor signs. A dense face-mesh (MediaPipe: 468 points) overlay provides stable geometric references for head-pose normalization and region-of-interest tracking. For privacy reasons, the subject photo has been generated with AI (DALL·E 3 model, OpenAI, San Francisco, CA, USA), and the landmarks have been subsequently computed with MediaPipe (MediaPipe, Google, 2023, Version 0.10.26, 10 July 2025).

Hypomimia is a reduction of spontaneous and voluntary facial movement, typically presenting as decreased blink rate and diminished lower/upper facial expressivity. The patients have to be observed at rest for ~10 s (without talking) and while talking; specifically note blink frequency, masked facies/loss of expression, spontaneous smiling, and parting of lips [2] (MDS-UPDRS-III item 3.2). CV enables objective, contactless quantification of oro-facial motor behavior from ordinary video. Dense facial landmarking (e.g., face-mesh or action-unit inference) and head-pose normalization allow extraction of amplitude, velocity, and temporal regularity of expressions (evoked and spontaneous), as well as asymmetry across the hemiface. Blink dynamics (rate, inter-blink interval, duration, habituation to glabellar taps) and jaw tremor can be derived from landmark trajectories; subtle oscillations are further detectable with EVM (Figure 9). These kinematic time series support computation of task-level features (e.g., smile aperture/velocity, freezing or delay of initiation) and continuous severity scores aligned with clinical items, using rule-based thresholds or supervised models.

- Diagnosis

An SVM algorithm leveraging facial expression amplitude achieved an F1 score of 99% for PD diagnosis [98].

Abrami and colleagues [99] developed a convolutional neural network to automatically detect hypomimia in PD from video recordings. The model was trained on images from 107 self-identified PD patients and 1595 control videos, then applied to clinical interview videos of 35 PD patients in on/off medication states and seven longitudinal interviews of actor Alan Alda before and after his PD diagnosis. On the test set, the algorithm achieved an AUROC of 0.71 for distinguishing PD hypomimia, comparable to expert neurologist ratings (AUROC 0.75). The model classified on/off drug states in clinical videos with 63% accuracy (versus 46% using rater scores) and perfectly distinguished pre- versus post-diagnosis in Alan Alda’s interviews (Table 5).

#### 3.5.2. Gait, Balance and Posture

On MDS-UPDRS Part III, gait (MDS-UPDRS-III item 3.10) is rated while the patient walks ≥10 m, turn and return, judging stride length/speed, foot strike, turning, and arm swing (freezing scored separately), 0–4 from normal to unable to walk without assistance. Freezing of gait (MDS-UPDRS-III item 3.11) is scored 0–4 based on halts at start/turns/doorways and during straight walking. Postural stability (item 3.12) is assessed with the pull test and scored 0–4 by the number of recovery steps and the need for assistance. Posture (MDS-UPDRS-III item 3.13) is rated 0–4 by the worst observed stoop/scoliosis/lean and whether it can be corrected voluntarily.

**Figure 10:**
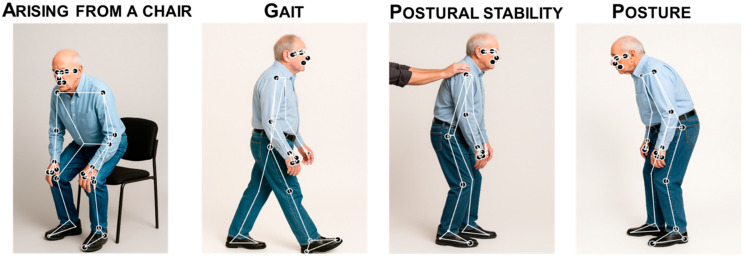
CV overlays for gait, balance, and posture assessment. From left to right: sit-to-stand initiation (hip–knee–ankle and trunk keypoints for rise time, peak velocity, and joint excursions), steady gait (stride/step length and time, cadence, stance–swing, arm-swing amplitude/asymmetry), pull test/perturbation (postural response latency, recovery steps, trunk angle), and posture (sagittal alignment/camptocormia angle). For privacy reasons, the subject photo has been generated with AI (DALL·E 3 model, OpenAI, San Francisco, CA, USA), and the landmarks have been subsequently computed with MediaPipe (MediaPipe, Google, 2023, Version 0.10.26, 10 July 2025).

CV enables contactless, scalable quantification of gait, balance, and posture from ordinary RGB video. Modern pose estimators extract 2D/3D joint trajectories from which spatiotemporal gait parameters are derived—speed, cadence, step/stride length and time, stance/swing, double support, arm-swing amplitude/asymmetry, and gait variability; cycle-wise regularity and inter-limb phase support the detection of freezing of gait and festination. In clinical tasks (over-ground walking, Timed Up-and-Go, sit-to-stand transitions), CV computes trial-level metrics such as turn duration/angle, rise time, and turning velocity. For balance, stabilizing the camera and tracking head–torso–pelvis landmarks allows estimation of center-of-mass proxies and postural sway in time and frequency domains (RMS, velocity, path length, ellipse area) during quiet stance or perturbations. Posture can be assessed from single frames or short clips by measuring sagittal/coronal alignment (camptocormia, Pisa syndrome) and joint angles under different loads. Monocular video supports broad deployment, while multi-view or depth cameras improve metric scaling and reduce occlusion (Figure 10). Combined with scene calibration (homography or fiducials) and model-based filtering, CV delivers rater-independent biomarkers suitable for telemonitoring, clinic triage, and therapy response tracking.

- Diagnosis

A pose estimation algorithm achieved 0.86 accuracy in differentiating PD from spinocerebellar degeneration using 2D video [92].

Quantitative assessment using 2D video revealed distinct gait characteristics in PD patients (shorter step length, faster cadence, slower walking speed, smaller arm-swing angle, increased variability, and asymmetry) compared to HC. A combined diagnostic sensitivity and specificity of 84.6% and 89.6%, respectively, and an AUROC of 0.91 were achieved for early-stage PD [100].

A volumetric deep architecture achieved 98% classification accuracy in distinguishing PD patients from control subjects based on gait patterns using OpenPose activations [101]. A monocular video 3D pose estimation method achieved 93.3% classification accuracy for early PD detection based on gait analysis [102].

Shah and colleagues [103] proposed a video-based deep learning model to classify PD using gait data recorded via smartphone cameras. They collected videos of 74 PD patients and 110 healthy controls performing a standardized 10-m walk, extracting 2D body keypoints with BlazePose. A convolutional neural network trained on the spatiotemporal sequences achieved high diagnostic performance (accuracy: 94.3%; AUC: 0.979).

Cao and colleagues [104] developed a video-based 3D-CNN to automatically detect shuffling steps in PD from TUG videos. Using RGB silhouettes cropped to legs/feet from 18 PD patients and 42 HC, a spatiotemporal feature extractor plus fusion module achieved 90.8% accuracy for shuffling detection and 84.2% for severity grading.

Lu and colleagues [105] introduced a 3D pose extraction from monocular videos using a neural model (SPIN), followed by severity classification via a temporal convolutional neural network to estimate gait severity scores according to the MDS-UPDRS in PD. The model achieved high classification performance, with an overall macro-average F1-score of 0.83, AUC of 0.90, precision of 0.86, and balanced accuracy of 81%.

An unsupervised deep learning pipeline quantified gait from standard home videos of PD and controls [106]. Using OpenPose to extract 2D joints, cadence was computed via short-time autocorrelation (ST-ACF). Cadence from frontal-view videos agreed with lateral recordings (R^2^ = 0.754).

In the work of Chavez and colleagues [107], videos of normal and Parkinsonian gait were analyzed through OpenPose to extract gait features such as stride length, step time, swing time, and double support. k-NN, SVM, and gradient boosting models were trained and achieved high classification accuracy, ranging from 96% to 99%.

Ripic and colleagues [108] validated the AI-based markerless system KinaTrax against a marker-based reference in 57 participants (23 young, 14 older, 20 PD; 216 trials). Using velocity- and coordinate-based event detection, most spatiotemporal parameters showed excellent agreement (ICC > 0.90); swing time was slightly lower (ICC≈0.88–0.91). Bland–Altman analysis indicated minimal bias (<1%) and narrow limits of agreement.

Simonet and colleagues [109] tested the markerless Theia3D system for mediolateral margin of stability (MoS) at foot contact on dual force plates during gait initiation in 12 PD, 10 young, and 11 older adults. MoS showed no system effect, with near-zero mean bias and 95% limits of agreement ≈ −0.02 to 0.03 m, supporting the method’s validity.

Multimodal approaches fusing eye and gait motion patterns using covariance descriptors have reported high accuracy (96.1%) for individual modalities [110] (Table 6).

- Symptom monitoring

OpenPose for 2D skeleton extraction and a spatiotemporal convolutional neural network achieved a sensitivity of 84%, a specificity of 96%, and an accuracy of 91% for FoG detection in 36 patients with parkinsonism. Cohen’s κ for agreement with clinical raters was 0.78 [111].

Rupprechter and colleagues [112] proposed an interpretable CV method to quantify PD gait impairment. From 729 gait videos, OpenPose keypoints yielded features (step frequency, arm swing, postural control, smoothness) fed to an ordinal random forest model predicting MDS-UPDRS 3.10. The model achieved 50% balanced accuracy, was within ±1 point of clinician ratings in 95% of cases, and correlated with ratings (ρ = 0.52, *p* < 0.001).

Khan and colleagues [113] developed a front-view, silhouette-based CV framework to classify UPDRS-gait severity from 456 videos of 19 PD patients, yielding stride length, shuffling, festination, and walking speed as features. An SVM distinguished three levels (healthy, mild, moderate) with 70.8% accuracy and an AUC of 0.81.

Li and colleagues [114] developed an automated, video-only pipeline to segment and time Timed Up-and-Go (TUG) sub-tasks in PD. From 127 videos of 24 patients, OpenPose extracted 2D keypoints; normalized joint trajectories fed SVM and LSTM classifiers to label frames into six TUG phases. The best model (OpenPose + LSTM) achieved 96.9% frame-level accuracy and precise sub-task timing.

Gait features calculated from 2D joint trajectories derived from video analysis have shown strong correlation with instrumented walkway data, indicating their suitability for longitudinal gait monitoring in PD patients [115].

A markerless, camera-agnostic method was developed to quantify axial postural abnormalities in PD by augmenting human pose estimation with clinically relevant keypoints (C7, L5, mid-ankle, and the fulcrum of maximal deviation). Tested on 76 RGB images from 55 patients spanning camptocormia and Pisa syndrome, the approach was robust and accurate across resolutions and subject–sensor distances, and its measurements showed no meaningful dependence on anthropometry or image properties [116].

Ling-Yan Ma and colleagues [117] developed vision-based, remote scoring models for parkinsonian rigidity and postural stability using a low-cost RGB camera and indirect MDS-UPDRS III tasks. In 104 PD patients, motion features extracted with OpenPose/HRNet/OpenFace fed LightGBM classifiers that estimated item scores, with the best performance for lower-limb rigidity (accuracy ≈ 70%, κ = 0.66, ρ = 0.76) and postural stability (accuracy ≈ 73%, κ = 0.66, ρ = 0.68).

Shin and colleagues [118] developed an automated approach to objectively evaluate postural abnormalities in PD using deep learning-based pose estimation. Lateral photographs of 28 PD patients were analyzed using the OpenPose algorithm to measure anterior flexion angle and dropped head angle. Results demonstrated excellent agreement with manual labeling methods (ICC > 0.95), with mean biases ≤ 3 degrees for both angles (Table 6).

**Table 6:** Summary of included studies focusing on gait, balance, and posture assessment classified according to PICO questions per PD patients and controls enrolled, pose estimation software, recording device, performance, and technology used for validation.

Aim	Ref.	Patients	Controls	Device	Pose Estimation	Performance	Technology Comparison
Diagnosis	[86]	40	37	Smartphone (1920 × 1080 px, 60 fps)	Victor Dibia Handtracking, preprocessing in OpenCV	Test accuracy 0.69, precision 0.73, recall 0.76, AUROC 0.76	NA
	[108]	20	37	KinaTra processed in Visual3D (KinaTrax Inc., Boca Raton, FL, USA)	KinaTrax HumanVersion3	Speed ICC 0.995, stride length ICC 0.992; swing time ICC ≈ 0.910. Bland–Altman speed bias 0.000 ± 0.020 m/s, LOA −0.039 to 0.038	Marker-based optoelectronic system (SMART-DX, BTS)
	[110]	13	13	Standard RGB camera (~520 × 520, 60 fps)	Dense optical	Early/late fusion (kinematic + deep) Accuracy 1.00, Sensitivity 1.00, Specificity 1.00 (leave-one-patient-out); best single modality: gait-DF (MobileNetV2 4th layer) accuracy 0.961, eye-KF accuracy 0.923	NA
	[100]	68	48	Smartphone videos (1080p, 30 fps)	OpenPose	AUC 0.91 Sensitivity 84.6% Specificity 89.6% Plantar-pressure insoles: (|Δ| = 0.056 s stance, 0.031 s swing, 0.037 s cycle) and 3D motion capture for spatial/upper-limb metrics (|Δ| = 1.076 cm step length, 0.032 m/s speed, 4.21° arm-swing angle, 9.06°/s arm-swing velocity)	Insole plantar-pressure
	[101]	14	16	RGB camera	OpenPose	Classification accuracy up to 99.4% AUC 0.999–1	NA
	[102]	34	36	RGB camera	OpenPose	Accuracy 95.8% Sensitivity 94.1% Specificity 97.2%	NA
	[119]	34	25	Single RGB camera (30 fps) synchronized with VICON motion capture (Vicon Ltd., Oxford, UK, 100 Hz) and AMTI force plates (AMTI Optima HPS, Watertown, MA, USA, 1 kHz)	HRNet + HoT-Transformer (3D reconstruction) + GLA-GCN (spatiotemporal modeling)	− ICC > 0.70 for most gait metrics; correlation r > 0.80 with VICON/AMTI; − PD vs. control: classification accuracy 93.3%	VICON (3D motion capture)
	[106]	119 (PD + HC)	119 (PD + HC)	Everio GZ-HD40 camera for PD; standard video camera from CASIA dataset for controls	OpenPose	Cadence estimation in controls: good agreement between frontal and lateral views (R^2^ = 0.754, RMSE = 7.24 steps/min, MAE = 6.05 steps/min). ROC AUC 0.980 for kNN in pre/post DBS comparison	NA
	[105]	30	20	Monocular clinic videos, ~30 fps	SPIN; SORT tracking; ablation with OpenPose (2D)	Macro-F1 0.83, AUC 0.90, precision 0.86, balanced accuracy 81%	NA
	[109]	12	21	Dual AMTI force plates (AMTI Optima HPS, Watertown, MA, USA) + markerless system: 12 Qualisys cameras (Qualisys, Göteborg, Sweden)	Theia3D v2021.2.0.1675	Bland–Altman: mean bias ~0; typical 95% limits about −0.02–0.03 m; BF_01_ = 7.4	Force plates (AMTI)
	[107]	1 PD source video for training + 1 early-PD video for external check	1 normal-gait video (treadmill)	RGB video camera	OpenPose	Multiclass stage classification accuracy—Gradient Boosting 0.99, KNN 0.97, SVM 0.96; classwise AUCs ~1.0 and F1 (0.94–0.97 overall)	NA
Symptom monitoring	[114]	36	-	Microsoft Kinect v2 depth camera (RGB + depth, 30 Hz, Microsoft corporation, Redmond, Washington, DC, USA)	Kinect SDK skeletal tracking	Sub-task segmentation accuracy 94.2%, with MAE in sub-task duration < 0.3 s	NA
	[118]	28	-	Smartphone camera (iPhone 6s, Apple Inc, Cupertino, CA, USA)	OpenPose	ICC > 0.95 for both AFA and DHA; mean bias ≤ 3°; vertical reference bias mean 0.91°	Manual labeling (stickers)
	[115]	25	-	Webcam (Logitech C920, 480 × 640@30 Hz, Logitech International S.A., Lausanne, Switzerland) + Zeno walkway (120 Hz, ProtoKinetics, Havertown, PA, USA) in clinic	OpenPose, AlphaPose, Detectron (2D); ROMP (3D)	2D video features show moderate–strong positive correlations with Zeno for steps, cadence, step-width mean/CV	Zeno instrumented walkway (PKMAS)
	[103]	24	-	8-camera OptiTrack mocap (240 Hz, NaturalPoint Inc, Corvallis, OR, USA) + 2 smartphone cams (Galaxy A Quantum, 30 Hz, Samsung Electronics, Samsung Digital City, Suwon, South Corea)	MediaPipe	Temporal MAE typically 0.00–0.02 s; treadmill step length MAE 0.01 m, speed MAE 0.03 m/s; overground (lateral) step length MAE 0.09 m (fwd)/0.07 m (back), speed MAE 0.17/0.13 m/s	3D motion capture (OptiTrack/Motive)
	[112]	729 videos	-	Smartphone/tablet recordings (KELVIN-PD™ app, Machine Medicine Technologies, London, UK)	OpenPose	Balanced accuracy 50% Spearman ρ = 0.52 with clinician ratings; step-frequency *r* = 0.80 vs. manual counts	NA
	[113]	456 videos from 19 patients	-	Single RGB camera, 25 fps, 352 × 288 px	HOG human detector	Accuracy 70.83%; AUC 0.81. Feature mean-ranks significantly separated severity levels (Kruskal–Wallis, *p* < 0.05)	NA
	[111]	9	-	Monocular RGB cam (Panasonic HC-V720M-T, 720 × 480, 30 fps, Panasonic Corporation, Kadoma, Japan)	Light track, MediaPipe	Model II (5-fold CV): Accuracy 93.2% Precision 97.9% Recall 88.8% Specificity 97.9% ICC 0.75–0.94	NA
	[116]	55	-	Smartphone	OpenPose BODY25 and AutoPosturePD	ICC vs. image width R = 0.46, *p* = 0.026; lCC vs. subject “cover factor” width R = 0.51, *p* = 0.0084, height R = −0.43, *p* = 0.027, area R = 0.43, *p* = 0.03; lCC error vs. image height R = −0.46, *p* = 0.019, cover width R = −0.43, *p* = 0.028, cover area R = −0.44, *p* = 0.024)	NA

Abbreviations: PD = Parkinson’s disease; HC = Healthy controls; NA = not assessed; fps = frames per second; px = pixels; NA = not assessed; MDS-UPDRS = Movement Disorder Society Unified Parkinson’s Disease Rating Scale; LOA = Limits of Agreement; EVM = Eulerian Video Magnification; MAE = Mean absolute error; ICC = Intraclass coefficient; KPCA = Kernel Principal Component Analysis; AFA = anterior flexion angle; DHA = Dropped Head Angle; FoG = Freezing of gait; AUC = Area Under the Curve; ROC = Receiver Operating Characteristic; TPR = True Positive Rate; SVM = Support Vector Machine; KNN = K-nearest neighbors; HOG = histogram of oriented gradients; DBS = Deep Brain Stimulation; DTW = dynamic time warping; CV = cross-validation.

### 3.6. MDS-UPDRS-III

#### Symptom Monitoring

Morinan and colleagues [120] developed a CV-based model to quantify whole-body bradykinesia in PD, using video recordings from 628 patients. OpenPose extracted kinematic features from video-recorded bradykinesia tasks. ML models predicted composite bradykinesia scores (sum of MDS-UPDRS-III items 3.4–3.8), achieving strong agreement with clinical ratings (ICC = 0.74, *p* < 0.001). Individual task ratings demonstrated acceptable accuracy (81%), and binary severity classification yielded an AUC-ROC of 0.81.

Park and colleagues [121] used OpenPose-derived kinematic features from videos of resting tremor and finger tapping in 55 PD patients to train an SVM for automated UPDRS scoring. The model showed excellent agreement with clinical ratings for resting tremor (κ = 0.791, ICC = 0.927) and good agreement for finger tapping (κ = 0.700, ICC = 0.793), outperforming nontrained raters for tremor and performing comparably for tapping.

He and colleagues [119] presented a CV-based framework for objective PD assessment using two-dimensional pose estimation from standard video recordings of multiple motor tasks. Extracted features showed strong correlations with clinical ratings from the MDS-UPDRS (|r| up to 0.83, *p* < 0.001), with ICC up to 0.91 and Cohen’s κ up to 0.88 for inter-rater agreement.

Sarapata and colleagues [122] developed a multi-site deep-learning pipeline to automatically recognize MDS-UPDRS III motor activities from video. Using 7310 clips from 1170 assessments, OpenPose keypoints were formed into spatiotemporal graphs and classified with ST-GCN, achieving 96.5% video-level accuracy and high ROI localization (IoU > 0.8).

Mifsud and colleagues [123] leveraged pose estimation on consumer-grade, non-standard videos of the finger-to-nose task to extract robust kinematic features and train SVM and random forest classifiers under home-like, unstructured recording conditions. The SVM achieved F1 = 0.93 and accuracy = 0.90, matching or surpassing methods that require controlled video acquisition or wearable sensors.

Liu et al. [124] developed a low-cost, single-RGB-camera pipeline to score MDS-UPDRS III bradykinesia. In 360 videos from 60 PD patients (finger tapping, hand opening–closing, pronation–supination), a lightweight pose estimator (~7 FPS) extracted hand trajectories; four kinematic features capturing amplitude, speed, and variability fed supervised classifiers to predict 5-point scores. The system achieved 89.7% accuracy, comparable to sensor-based methods.

Xu and colleagues [125] developed a CV-based system to automate motor severity assessment in PD. In a multicenter study, 128 patients underwent MDS-UPDRS Part III video examinations, which were rated by expert clinicians and analyzed using pose estimation and ML. The model achieved high agreement with expert consensus (absolute accuracy: 69.6%; acceptable accuracy within ±1 point: 98.8%) and outperformed inter-rater reliability (MAE: 0.32 vs. 0.65).

A deep learning framework using OpenPose and BiGRU models quantified motor severity in PD based on video recordings of gait, leg agility, and toe tapping. In 40 PD patients and 30 healthy controls, features extracted from joint trajectories were correlated with MDS-UPDRS III scores for corresponding tasks. Strong correlations were found for gait (*r* = 0.82), leg agility (*r* = 0.75), and toe tapping (*r* = 0.71) [93] (Table 7).

### 3.7. Video Analysis Algorithms for PD Diagnosis: A Categorized Overview

Pose estimation software for diagnostic aims (PD vs. HC) was synthesized by type of Input/Representation keypoints (Table 8). This categorization makes explicit the translational mapping between input representation and the motor task assessed, indicating which modalities are fit-for-purpose and the corresponding clinical endpoints. Main categories were 2D key-points, 3D pose, Silhouette/Optical-flow/Motion, and raw video.

Two-dimensional keypoint pipelines predominate and are typically paired with either feature-based classical ML or end-to-end deep models, often relying on OpenPose/MediaPipe or custom CV front ends. Three-dimensional pose approaches are increasingly represented, frequently coupled with sequence/graph models operating on skeleton trajectories, whereas Face/Eye features (e.g., hypomimia/blink) and Silhouette/Optical-flow/Motion cues appear less commonly. Across categories, gait remains the dominant clinical target, with fewer studies focused on bradykinesia, tremor, or face expressions. Moreover, across studies leveraging keypoint-derived inputs, diagnostic performance was robust—especially for gait (AUC 0.91–0.99; accuracy 86–100%)—with wider variability for bradykinesia (accuracy 75–95%; AUC up to 0.97) and mixed results for facial-expression tasks (AUC 0.71 to F1 0.99).

### 3.8. Therapeutic Management

**Figure 11:**
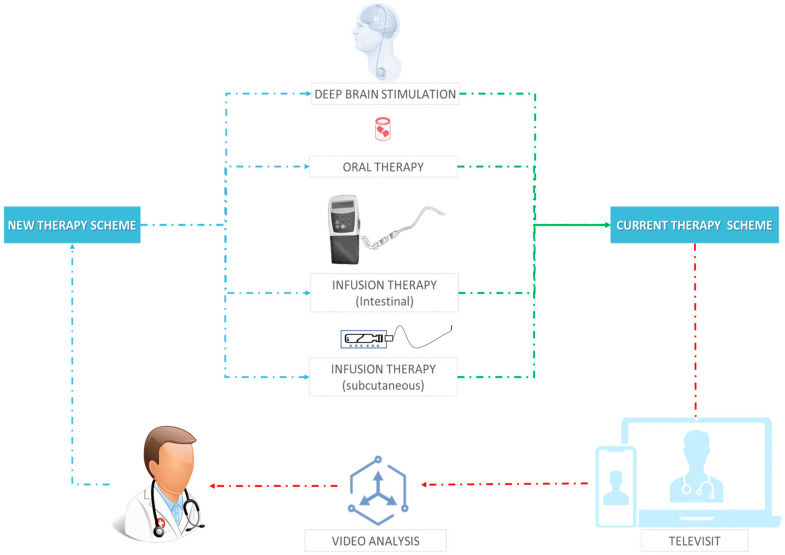
Workflow for computer-vision–enabled therapy management in PD during televisits. Green dash-dotted arrows: current therapy scheme directing patients to available options (deep brain stimulation, oral therapy, intestinal infusion, subcutaneous infusion). Light-blue dashed arrows: proposed new therapy scheme indicating potential treatment selection or adjustment. Red dashed arrows: telemedicine/data workflow linking Televisit, Video analysis, and the Clinician. Arrowheads indicate the direction of information or clinical-decision flow (where drawn in both directions, communication is bidirectional). Boxes denote treatment modalities; icons illustrate the delivery mode.

CV can allow therapy management in PD through objective kinematic parameters extracted from patient videos. In the illustrated pipeline (Figure 7), videos captured during in-person or remote visits, or recorded by patients between visits, can be analyzed, and the resulting severity scores and longitudinal trends are returned to clinicians. These data support titration of the current therapy scheme (e.g., timing and dose of oral levodopa, optimization of intestinal or subcutaneous infusion parameters) and provide quantitative endpoints for device programming (Deep Brain Stimulation, DBS amplitude/frequency/contact settings). When motor fluctuations, refractory OFF periods, or troublesome dyskinesias are detected, CV metrics can trigger algorithmic alerts and structured triage to consider advanced therapies (DBS or infusion therapies) as part of a new therapy scheme. Since analysis is contactless and reproducible, the same metrics can be used for remote follow-up, comparing ON vs. OFF states, documenting response to changes, and shortening iteration cycles between televisits and in-person care. Overall, CV-enabled video analysis can complement clinical judgement by delivering continuous, standardized measurements that close the loop between observation, decision, and therapy adjustment (Figure 11).

#### 3.8.1. Motor Fluctuations

CV measures of speed, amplitude, and rhythm correlated significantly with clinical ratings (*r* = 0.740 for MDS-UPDRS) and could classify medication ON/OFF states as accurately as human raters [89].

Chen and colleagues [126] introduced a monocular CV pipeline that extracts silhouettes and applies kernel PCA to derive spatiotemporal gait features. In 12 PD (OFF/ON) and 12 controls, it estimated cycle time, stride length, velocity, and cadence, achieving 80.5% classification accuracy. Metrics were levodopa-responsive (improved stride length and step frequency), and spectral analysis showed slower, more irregular, lower-frequency gait in the Drug-Off state.

Morgan and colleagues [127] validated a fully automated, video-based method to quantify sit-to-stand (STS) transitions from 85 h of home footage (12 PD, 12 controls) using OpenPose-derived head trajectories. Automated STS duration and ascent speed correlated with manual timing (duration: ρ = 0.419, *p* = 0.042; speed: ρ = −0.780, *p* < 0.001) and with MDS-UPDRS III (duration: ρ = 0.457–0.464, *p* < 0.05; speed: ρ = −0.691 to −0.723, *p* < 0.01). Group comparisons showed longer duration and slower ascent speed in PD ON medication versus controls (duration: U = 6263, *p* = 0.018; speed: U = 9965, *p* < 0.001), and slower speed OFF versus ON (*p* = 0.016) (Table 8).

#### 3.8.2. LIDs

A vision-based system quantifying changes in dyskinesia demonstrated sensitivity and specificity similar to or superior to the UDysRS, with an AUROC of 0.822 for onset detection and 0.958 for remission detection [128].

Li and colleagues [129] involved nine PD patients with LIDs undergoing a levodopa infusion protocol, with regular clinical assessments using UDysRS and UPDRS. Videos of motor tasks were analyzed using Convolutional Pose Machines to extract joint trajectories. Kinematic and frequency features were used to train random forest models for detecting and rating the severity of parkinsonism and LID. For LID, the communication task achieved the best performance (AUC = 0.93; severity correlation *r* = 0.661), while for parkinsonism, leg agility yielded the best severity correlation (*r* = 0.618) and toe tapping the best detection accuracy (AUC = 0.773). Combined task features predicted total UDysRS and UPDRS Part III scores with *r* = 0.741 and *r* = 0.53, respectively (Table 8).

#### 3.8.3. Therapy Response

Vision-based models have been evaluated for their ability to identify changes in predicted parkinsonism scores within individuals in response to medication and DBS [130].

In a retrospective analysis of 61 videos from 32 patients with PD, a video-based tracking algorithm detected antiparkinsonian effects: In the ON state, step length and gait velocity increased, while turning steps and turning time decreased (all *p* < 0.001). Improvements were consistent across individuals and evident on paired review. Cadence and step-length variability were unchanged, consistent with prior evidence that these metrics are relatively dopaminergic-insensitive [131].

Deng and colleagues [132] analyzed smartphone/tablet videos to quantify motor severity and treatment state in parkinsonism. In 31 patients, 3–7 s clips of gait and finger tapping were processed with MediaPipe to extract body/hand landmarks and temporal–spectral kinematics. Fusing body + hand features classified higher vs. lower MDS-UPDRS III with AUC 0.79 and ~72% accuracy; body-only reached an AUC of 0.76, hand-only ~0.69.

Automated analysis of hand movements using high-frame-rate videos and MediaPipe successfully quantified the effect of standard oral treatments, showing a decrease in mean frequency and amplitude after medication in PD patients [133].

A high-frame-rate (≥180 fps) video tool using MediaPipe Face Mesh was developed to quantify the glabellar tap reflex and blinking in 11 idiopathic PD (iPD) and 12 HC. Reflex blinks were defined as occurring 50–200 ms post-tap. iPD showed no habituation pre/post-dopaminergic therapy, whereas HC habituated after four taps (*p* < 0.001). iPD exhibited higher frequency and longer duration of non-reflex blinks; medication reduced frequency (*p* = 0.0026) but not duration (*p* = 0.6943) [134].

Using an SVM trained on extracted kinematic features, motor behaviors were categorized with an overall accuracy of 85.7%, including 92.3% for arm chain pulls and 76.2% for hand clenches. Leave-one-out validation across patients confirmed consistent classification performance (91.5–94.5%) [135] (Table 9).

### 3.9. Quality Appraisal

Authors substantially agreed that included studies had a low risk of bias for patient selection (69% low, 31% unclear), index test (71% low, 29% unclear), reference standard (72% low, 28% unclear), and patient flow and intervention timing (70% low, 30% unclear). Nonetheless, the authors identified for the majority of works low concerns regarding applicability to the research question for patient selection (80% low, 20% unclear) and reference standard (80% low, 20% unclear) domains, and concordantly low concerns regarding applicability for the index test (100% low). A checklist of quality metrics of included studies is summarized in Table S1, while Figure S1 depicts a graphical representation of mean values of all authors’ scores, from low to high, of QUADAS-2 items for risk of bias and concerns regarding applicability to the review question according to authors’ judgment.

## 4. Discussion

This systematic review examines the application of CV technology for different motor tasks for PD diagnosis, symptom monitoring, and therapeutic management.

Our review advances a clinician-first perspective beyond engineering-oriented surveys by organizing evidence around actionable motor symptoms and clinical use-cases (diagnosis vs. monitoring), privileging bedside-relevant endpoints (task-level accuracies, agreement with reference systems), and external validity over model architectures. In doing so, it directly addresses implementation questions—what a clinician can measure, with which setup, and under what constraints—that are often peripheral in engineering-oriented reviews.

In this regard, one limitation of the present review is that we limited database searching to PubMed to target clinician-anchored studies and endpoints. Other potentially relevant papers were captured through backward/forward citation chaining from included PubMed records and prior reviews.

The findings from the included studies strongly support the utility of CV technologies in quantifying motor symptoms of PD, in line with previous literature on the topic [58]. In this systematic review, we synthesized 45 original research articles applying CV technologies to PD, spanning across three aims: diagnosis, symptom monitoring, and therapy management. Of these, 44% (N = 20) focused on diagnostic purposes, using CV to distinguish PD patients from healthy subjects or other movement disorders based on single-timepoint motor assessments. 35% of studies (N = 16) addressed telemonitoring of motor symptoms, leveraging longitudinal or real-world video recordings to assess disease severity or track motor symptoms over time. The remaining 21% (N = 9) studies evaluated therapy-related effects, including the quantification of medication ON/OFF states, LIDs, or therapy responses. Across the studies included in this review, the most commonly analyzed motor task was gait, assessed in 42% (N = 19), while bradykinesia was the second most frequent task, featured in 17.7% of studies (N = 8), primarily for quantifying finger tapping and classifying disease severity. Less frequently investigated tasks included multitask paradigms to estimate MDS-UPDRS-III (N = 4, 8.8%), tremor assessment (N = 3, 6%), and facial expression analysis (N = 2, 4%). Finally, 22% (N = 10) of included studies investigated CV application for therapeutic management, including motor fluctuations, LIDs recognition, and therapy response.

**Figure 12:**
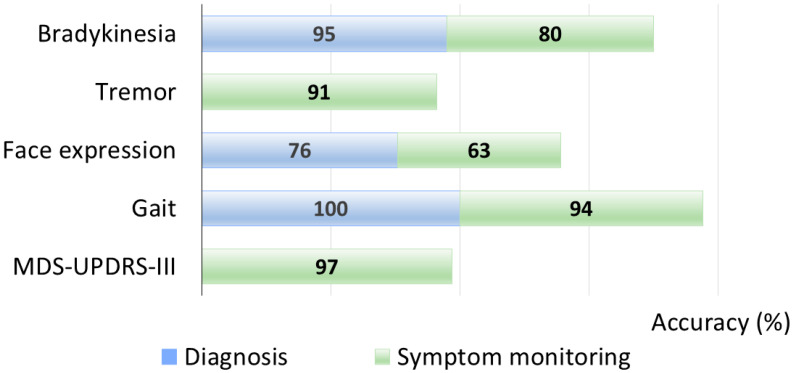
Best-case accuracies from the included CV studies by motor domain and aim. Bars show the highest reported accuracy for diagnosis (blue) and symptom monitoring (green).

This distribution highlights the predominance of gait-related applications within the current CV literature on PD, likely reflecting both the clinical relevance of gait impairment and the feasibility of extracting reliable kinematic features from video recordings. Finger tapping also emerged as a preferred task for bradykinesia quantification due to its standardization within clinical rating scales and suitability for short, camera-facing recordings. Finally, a clear gradient in CV performance exists across motor domains (Figure 12).

Gait yielded the highest diagnostic performance (reported at 100%) and remained strong for symptom monitoring (94%), reflecting the robustness of spatiotemporal gait features captured from whole-body pose. Video-based MDS-UPDRS III estimation achieved the best monitoring accuracy (97%), consistent with standardized task sets and multi-item aggregation, which is a more reliable severity classifier than a single-item binary detector. Tremor monitoring performance was also high (91%), as frequency and amplitude are well suited to video extraction and can be enhanced by motion magnification, whereas diagnostic use is less represented. Bradykinesia analysis performed well for diagnosis (95%) but dropped for monitoring (80%), likely due to task heterogeneity and different protocols. Facial expression analytics is promising (76% diagnosis; 63% monitoring) but still sensitive to mood, lighting, and head pose.

**Figure 13:**
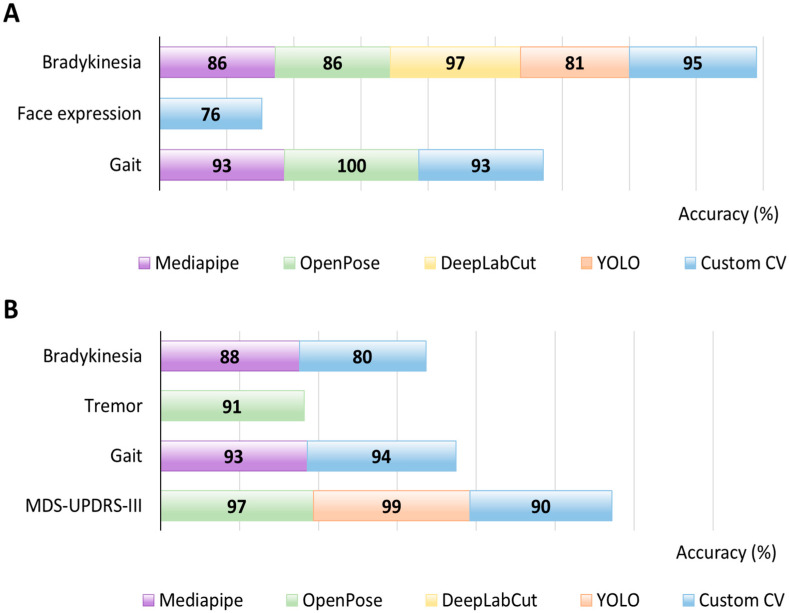
Best-reported accuracies by pose-estimation models across tasks. (**A**) Diagnosis. (**B**) Symptom monitoring.

Moreover, we classified pose estimation models based on their performance: for diagnosis, gait attains a near-ceiling result with OpenPose (100%), while bradykinesia peaks with DeepLabCut (97%) and a custom CV pipeline (95%). Evidence for facial expression in diagnostic classification is limited to a single custom solution (76%), indicating a less mature evidence base (Figure 13A). For symptom monitoring, MDS-UPDRS-III activity recognition yields the highest values (YOLO, 99%; OpenPose, 97%), gait remains strong (custom, 94%; MediaPipe, 93%), tremor is moderate (OpenPose. 91%), and bradykinesia shows the lowest range (MediaPipe, 88%; custom, 80%) (Figure 13B).

Importantly, across the 45 included studies, 24% (N = 11) performed head-to-head validation of CV outputs against an external technology reference system. For bradykinesia, video-derived tapping traces were benchmarked against a marker-based high-speed video (HSV) system, showing near-overlapping waveforms with mean peak/valley errors of ~3.3/2.4 px, respectively [88]. For tremor, smartphone video agreed closely with concurrent accelerometry (MAE, 0.10 Hz; 97% of estimates were within ±0.5 Hz of the accelerometer; near-zero Bland–Altman bias) [96].

Gait validations spanned multiple works: 3D motion capture and force plates (VICON/AMTI) with ICC > 0.70 for most metrics and *r* > 0.80 [119]; a markerless CV system (KinaTrax) against a marker-based optoelectronic setup (SMART-DX) with ICC > 0.90 for most spatiotemporal parameters, with minimal bias [108]; and dual force-plate assessment of mediolateral margin-of-stability with ~0 mean bias and 95% limits ≈ −0.02 to 0.03 m [109]. Against instrumented walkways, markerless video analysis showed moderate-to-strong correlations with Zeno/PKMAS features and high agreement with GAITRite for step length, velocity, and cadence (ICC > 0.90), albeit weaker for step-length variability (ICC ≈ 0.62) [115,131]. Additional comparisons reported small temporal MAE (~0.01–0.02 s) and spatial errors (~1 cm treadmill step-length MAE) versus OptiTrack mocap [103], and concordance of temporal events with plantar-pressure insoles (|Δ| ≈ 0.03–0.06 s), alongside centimeter-level spatial agreement with 3D motion capture for upper-limb metrics [100]. For axial posture, automated angles from smartphone video closely matched sticker-based manual measurements (ICC > 0.95; bias ≤ 3°) [118].

Overall, whole-body tasks currently provide the most robust CV readouts, while face metrics need further maturation in larger, prospective, externally validated cohorts.

### 4.1. Limitations of the Evidence and Research Gaps

Cited studies are constrained by small, often single-center samples; heterogeneous tasks, endpoints, and clinical anchors; and inconsistent reporting of acquisition parameters (camera model/frame rate, calibration) and preprocessing. Benchmarking against accepted reference systems is sporadic, while validation is predominantly internal, with limited multisite testing and insufficient analysis of domain shift. Ground-truth reliability (inter- and intra-rater) is rarely reported; longitudinal designs establishing predictive value and minimal clinically important change are uncommon. Implementation aspects—automated quality control, fail-safe pathways, clinician workload, patient acceptability, privacy-preserving deployment, and cost-effectiveness—are underexplored. Few papers investigate model performance across demographics or disease subgroups, limiting fairness assessment, and reproducibility remains a concern due to scarce code/data sharing and non-standardized feature-extraction processing.

Moreover, the predominance of small, single-center cohorts and reliance on internal cross-validation raise a substantive risk of overfitting, particularly when non-independent samples (frames, clips, repeated trials) are split across training and test sets, or when model selection/hyperparameters are tuned on evaluation data. Under these conditions, performance estimates likely overstate true generalizability. We also acknowledge the likelihood of publication bias and selective reporting; although a formal assessment was not possible due to heterogeneous metrics, small-sample positive findings should be interpreted cautiously. Crucially, external validation remains limited. Multisite studies that span different cameras, frame rates, lighting, and clinical populations—with subgroup/fairness analyses, drift monitoring, and open materials—are needed to demonstrate robustness and support clinical adoption.

Future work should adopt subject-independent splits, nested cross-validation, and report confidence intervals, calibration, and decision-relevance; pre-registration and prospective power justification can further reduce analytic flexibility.

### 4.2. From Bench to Bedside: Choosing CV Tools for Routine Care

In routine care, the choice of a CV pipeline should follow the clinical objective, with camera requirements scaled to the temporal precision needed.

For multi-joint tasks (gait spatiotemporals, sit-to-stand transitions, posture angles), 2D pose estimators, such as OpenPose or MediaPipe, offer a strong balance of accuracy, robustness, and deployment simplicity. OpenPose remains a solid option when richer skeletal coverage is required, whereas MediaPipe is lightweight and well-suited to real-time use on standard laptops or smartphones.

For fine, repetitive movements (finger tapping, rapid alternating movements), both OpenPose and MediaPipe work well; accuracy improves when paired with ≥60–120 fps capture to stabilize inter-tap timing and amplitude-decrement metrics.

For postural and resting tremor, frameworks that run efficiently at high frame rates (e.g., MediaPipe for keypoints) can be combined with simple signal processing on tracked Regions of Interest (ROIs). In research-grade contexts, DeepLabCut is advantageous because it allows user-defined keypoints and frame-by-frame labeling for precise amplitude/frequency estimation—at the cost of higher setup/training overhead.

For dystonia (segment-specific postures and patterned movements), DeepLabCut can target disease-salient landmarks (e.g., cervical alignment, hand postures) when generic skeletons underperform; for routine clinics, a pragmatic approach is MediaPipe/OpenPose with task-specific angles or symmetry indices.

For hypomimia/blink and oculomotor cues, face/landmark toolkits (MediaPipe Face, OpenFace) can be combined with validated timing features; if reflex latencies are endpoints, increase fps (≈180 fps) rather than model complexity.

3D pose (e.g., lifting 3D from 2D keypoints or RGB-D sensors) should be dedicated for endpoints that truly require absolute joint angles or out-of-plane motion (eg, ankle/foot kinematics, complex turns with occlusions); otherwise, 2D pipelines are usually sufficient and easier to maintain.

Finally, lightweight models (MediaPipe) are preferable for real-time clinics/telemedicine and customizable frameworks (DeepLabCut) for research or deep kinematic phenotyping; offline/on-device processing is mandatory when privacy is critical (face analysis); and acquisition should be standardized (stable mount, distance, lighting, full-ROI visibility) before increasing model complexity.

The take-home message is to consider the clinical objective when choosing CV for PD: It is essential to adopt a strategy tailored to the specific motor symptom and intended analysis. For example, the same movement disorder may require distinct methodologies if the clinical aim changes, in order to align the choice of pose estimation model with the motor features under investigation and the desired outcomes.

### 4.3. Opportunities and Challenges

CV provides an automated, quantitative, and scalable opportunity to evaluate motor symptoms, addressing several longstanding limitations of current clinical practice [136]. Conventional rating scales, such as the MDS-UPDRS, are time-consuming and rely on semi-quantitative, observer-dependent assessments that are prone to inter- and intra-rater variability [11,12]. In contrast, CV techniques generate reproducible, quantitative, and granular measurements of movement, thereby reducing subjectivity.

CV systems could accurately classify motor states or disease presence, sometimes achieving performance metrics comparable to, or even surpassing, human raters, at minimum time and resource expense. Unlike wearable sensors, which must be attached to the body and can subtly alter natural motor patterns, camera-based computer vision (CV) captures movement externally, enabling patients to perform tasks freely in ecologically valid, day-to-day settings [26,28,40,49]. However, home-collected signals are not automatically reliable: without rigorous quality control and appropriate statistical modeling, motor characterization may be biased or unstable [26,57]. Moreover, inferring true bradykinesia from sensor readouts alone can be misleading, as nonspecific slowness due to fatigue, anxiety, or fear of falling may mimic the phenotype and requires contextual information and clinical adjudication [28].

With the ability to utilize standard consumer devices such as smartphones, tablets, or webcams, CV can be deployed outside of specialized clinical environments, supporting remote assessments and enabling telemedicine-based care, especially in unserved areas [25,58,85]. This facilitates frequent monitoring of symptom fluctuations, enhances the detection of motor complications, and supports real-time treatment adjustments. Commodity smartphones can yield clinically usable signals when paired with lightweight guidance and automated quality checks, and in this regard, a video-based guide for at-home MDS-UPDRS-III has demonstrated usability above benchmark, supporting telemedicine workflows [137].

However, practical barriers that condition use in naturalistic conditions include camera geometry and positioning (frontal/lateral view, distance, field of view), lighting/background variability, occlusions and clothing, as well as the need for basic calibration/synchronization and patient adherence to standardized tasks. These factors materially affect pose tracking and generalizability and require simple mitigations, like brief recording instructions, automated quality-control checks for viewpoint/illumination/task completeness, and site standard operating procedures [58,82,85]. Collectively, this framing clarifies near-term translational pathways and the operational safeguards needed for reproducible in-clinic and at-home, real-world application.

**Figure 14:**
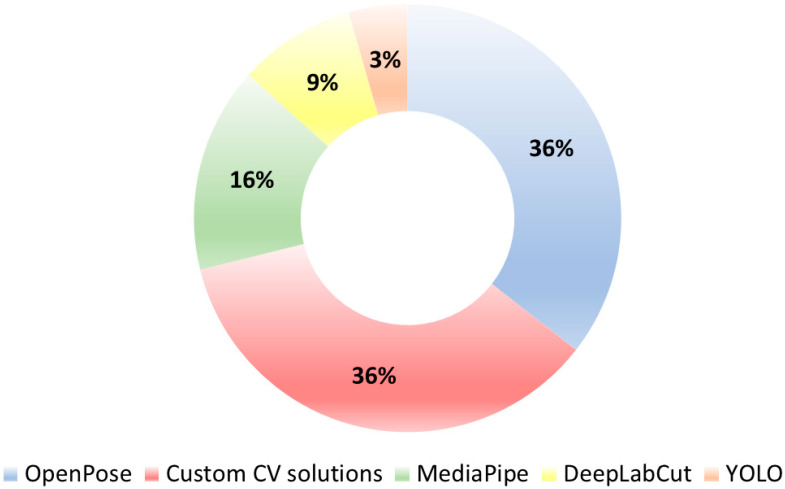
Relative representation of pose estimation software across included studies.

While the use of CV in PD assessment shows considerable promise, several challenges must be overcome to enable its widespread adoption in clinical practice. A major obstacle is the heterogeneity and lack of standardization across studies. There is significant variability in how video data are acquired and subsequently analyzed by software tools. Heterogeneity in videoanalysis protocols and feature extraction/selection is supported by our findings: OpenPose and custom CV solutions were reported as the most frequently used pose estimation software (36% each), followed by MediaPipe (16%), DeepLabCut (9%), and YOLO (4%) (Figure 14).

This inconsistency limits the reproducibility and comparability of findings, ultimately hindering the development of universally applicable models. As a result, generalizing findings across centers or patient cohorts becomes difficult, and efforts to develop clinical-grade tools may be delayed by these discrepancies. Moreover, simplifying video-derived data into one-dimensional time series could potentially discard rich spatial and temporal information. Although more sophisticated approaches—such as 3D pose estimation and deep learning directly applied to raw video—are now emerging, there remains no consensus on which features are most informative for characterizing PD motor symptoms. This step is critical, as the quality and relevance of extracted features directly influence the accuracy and interpretability of downstream ML models. Indeed, many of the high-performing CV pipelines rely on complex deep learning architectures that function as “black boxes,” offering limited transparency into how decisions are made. For clinicians aiming to adopt these technologies with confidence, it is essential that models provide interpretable outputs—highlighting which features contribute most to specific predictions, and why.

Ongoing efforts to integrate explainable AI methods are promising, but more work is needed to ensure these tools meet the demands of clinical decision-making [57,138].

Finally, while many studies report high classification accuracies within their datasets, it is unclear how these models perform across diverse populations, varying stages of disease, or real-world settings with uncontrolled lighting and camera angles. Robust performance under such conditions is essential for real-life applications—particularly in home-based or telemedicine contexts. Building models that maintain accuracy across a wide range of scenarios is a critical next step for real-world translation [26,57,58].

### 4.4. Future Directions

Moving from promising prototypes to routine care will require clinically oriented advances on several fronts. A critical gap emerging is the absence of a publicly available, clinically curated video dataset specific to PD suitable for benchmarking CV. Progress will require an open PD dataset with standardized tasks, harmonized recording conditions (camera geometry, distance, viewpoint, lighting), and clinically meaningful labels (item scores, disease stage, medication state), coupled with clear governance for privacy, consent, and data sharing. Establishing such a resource would enable reproducible comparisons across methods, accelerate external validation, and support translation into routine clinical and home-based assessment.

Clinical deployment should adhere to data-protection by design: explicit consent and purpose limitation; data minimization (short clips, task-only views); on-device preprocessing where feasible (pose extraction with immediate deletion of raw video); and de-identification (face blurring, pseudonymous IDs, removal of audio). All transfers must use end-to-end encryption; storage requires encryption at rest, role-based access control with least privilege, multi-factor authentication, audit logs/SIEM, and key management segregated from application services with rotation policies. Governance should follow GDPR/HIPAA-aligned policies, including a Data Protection Impact Assessment (DPIA), clear controller/processor delineation, data processing/use agreements, and predefined retention/deletion schedules per dataset and task; for cross-border transfers, appropriate safeguards and data localization should be specified when required. Research and clinical pipelines should document provenance (dataset versions, model versions, parameters), support independent auditing, and maintain incident-response/breach notification procedures with defined timelines. User rights (access, rectification, erasure, consent withdrawal) must be operationalized with workflows that remove or render unusable linked data and models that could re-identify subjects. For telemedicine scenarios, device hardening, ephemeral local caches, network segmentation, and automated quality-control checks (viewpoint, illumination, task completeness) need to be enforced before data upload. Where appropriate, privacy-preserving approaches (on-device analysis, federated learning) are preferable to reduce movement of raw video.

Because telemedicine is central, home-based validation must test agreement with in-clinic assessments, reliability, adherence, and automated quality control (viewpoint, illumination, task completeness), while addressing privacy and consent.

Notably, PD exhibits significant inter- and intra-patient variability in symptom expression, encompassing both motor and non-motor domains. Beyond video alone, future work should explore multimodal acquisition—combining large amounts of data from different platforms. In the future, raw video data could be integrated with inertial (e.g., IMUs, plantar-pressure insoles, instrumented walkways), autonomic (smartwatch for heart rate, oxygen pulse rate, blood pressure variation), and contactless radar (UWB or mmWave) data to recover occluded kinematics to enhance robustness across environments. Such fusion will require standardized synchronization and metadata schemas, interoperability across platforms, cross-sensor calibration, and methods that quantify and propagate uncertainty across modalities, ideally with lightweight, edge/on-device processing to protect data [40,57].

Finally, moving from prototypes to routine clinical and home use requires longitudinal and implementation studies that integrate model outputs into telehealth systems, validate digital endpoints for monitoring progression and treatment response, and assess cost-effectiveness and impacts on clinical workflow.

## 5. Conclusions

This systematic review overviews the state of the art on markerless video-based technology as an effective non-invasive tool for a quantitative assessment of PD. CV technologies present a transformative opportunity for the objective characterization of motor symptoms in PD. While promising, future research must prioritize standardization, data integrity, model transparency, and external validation. Addressing these challenges will be essential to ensure that CV technologies can be reliably integrated into routine clinical workflows and ultimately improve patient care.

## Figures and Tables

**Table 1:** Human pose datasets.

Dataset	Year	Size (Images/Frames)	Persons/Poses	Keypoints	2D/3D	Notes
COCO Keypoints	2014	200k+ images	250k persons	17	2D	Most widely used benchmark
MPII Human Pose	2014	25k images	40k persons	14–16	2D	Rich activity diversity
Human3.6M	2014	3.6M frames	11 subjects	32 (3D joints)	3D	Motion capture accuracy
CMU Panoptic Studio	2015	Multi-view recordings, 65+ HD cams	Thousands	Full body, hands, face, feet	2D and 3D	Enabled OpenPose full-body estimation
AI Challenger	2017	300k images	700k persons	14	2D	Large scale
CrowdPose	2018	20k images	80k persons	14	2D	Focus on occlusions
PoseTrack	2018	23k frames (video)	150k poses	15–17	2D	Tracking across time
COCO DensePose	2018	Subset of COCO	50k persons	Dense surface	2D→3D	Dense mapping
COCO WholeBody	2020	-	-	133 (17 body, 6 feet, 68 face, 42 hands)	2D	Whole-body keypoint annotations; extension of COCO Keypoints

**Table 2:** Comparative overview of major frameworks for pose estimation.

Framework	Year	Origin/Developer	Main Training Dataset(s)	Landmarks			2D/3D	Notes
				B	H	F		
OpenPose	2017	CMU (Pittsburgh, PA, USA)	COCO, MPII, CMU Panoptic	15, 18, 25	21	70	2D (3D with multi-view)	First real-time multi-person framework
Posenet	2017	Google AI (Mountain View, CA, USA)	COCO	17	-	-	2D	Real-time in-browser (TensorFlow.js); single-person
AlphaPose	2017	SenseTime (1900 Hongmei Road Xuhui District, Shanghai, China)	COCO, MPII	17	-	-	2D	Top-down, high accuracy multi-person
HRNet	2019	Microsoft (Redmond, WA, USA)	COCO, MPII	17	-	-	2D	Maintains high-res features
MediaPipe/BlazePose	2019–2020	Google (Mountain View, CA, USA)	COCO + internal + GHUM	33	21	478	3D (x,y,z)	Real-time, lightweight, GHU-based
YOLO-Pose	2022	Ultralytics (Frederick, MD, USA)	COCO keypoints	17	-	-	2D (3D experimental)	Extension of YOLO detectors
DeepLabCut	2018	Mathis Lab (Swiss Federal Institute of Technology, Lausanne (EPFL), Switzerland)	User-defined	Flexible			2D and 3D	Customizable, used in neuroscience
EfficientPose	2020	Megvii (27 Jiancheng Middle Road, Haidian District, Beijing, China)	COCO	17	-	-	2D	Efficiency-focused
Movenet	2021	Google AI (1600 Amphitheatre Pkwy, Mountain View, CA, USA)	COCO	17	-	-	2D	Lightning (low-latency) and Thunder (higher-accuracy) variants
DensePose	2018	Facebook AI (1 Hacker Way, Menlo Park, CA, USA)	COCO Dense	Dense surface mapping			2D → 3D *	Pixel-to-surface mapping

Abbreviations: B = Body; H = Hand; F = Face; CMU = Carnegie Mellon University. * = single-view lifting from RGB/2D keypoints to 3D pose/mesh; results are camera-frame and up-to-scale unless calibrated; no depth/multi-view.

**Table 5:** Summary of included studies focusing on facial expression analysis, classified according to PICO questions per PD patients and controls enrolled, pose estimation software, recording device, performance, and technology used for validation.

Aim	Ref.	Patients	Controls	Device	Pose Estimation	Performance	Technology Comparison
Diagnosis	[98]	33	31	Canon 700D on tripod (Canon Inc, Tokyo, Japan)	Face++ API	SVM F1 0.99 Precision 0.99 Recall 0.99 LR/RF F1 0.98; DT F1 0.93 LSTM Precision 0.86 Recall 0.66, F1 0.75	NA
	[99]	− Training: 107 PD videos; − Test set: 27 PD; − Clinical validation: 35 PD; − Longitudinal case: 1 PD (Alan Alda)	Training: 1595 control subjects (YouTube Faces DB, 3425 videos). Test set: 27 controls (54 total)	Microsoft Kinect camera at 30 fps (Microsoft corporation, Redmond, DC, USA)	Deep convolutional neural network (VGG-style)	− Diagnosis: AUROC 0.71 (algorithm) vs. 0.75 (neurologist) − ON vs. OFF: classification accuracy 63% for the algorithm vs. 46% using clinician score differences; per-session PD detection 76% (OFF) and 67% (ON) by the algorithm; clinical-interview ON/OFF accuracy 55% (algorithm) vs. 45% (neurologist) − Longitudinal validation: 7/7 Alan Alda interviews correctly classified	NA

Abbreviations: PD = Parkinson’s disease; HC = Healthy controls; fps = frames per second; MDS-UPDRS = Movement Disorder Society Unified Parkinson’s Disease Rating Scale; EVM = Eulerian Video Magnification; LR = Logistic Regression; RF = Random Forest; AUROC = Area Under the Curve Receiver Operating Characteristic; SVM = Support Vector Machine; DT = Decision Tree; LSTM = Long Short-Term Memory; VGG = Visual Geometry Group.

**Table 7:** Summary of included studies focusing on MDS-UPDRS-III assessment classified according to PICO questions per PD patients and controls enrolled, pose estimation software, recording device, performance, and technology used for validation.

Aim	Ref.	Patients	Controls	Device	Pose Estimation	Performance	Technology Comparison
Symptom monitoring	[123]	28	-	Handheld smartphone videos (30 fps)	PIXIE	− Finger-to-nose SVM F1 0.93, AUROC 0.94; RF F1 0.85. − Pronosupination SVM F1 0.20 RF 0.33	NA
	[122]	7310 videos from 1170 PD	-	Consumer mobile devices via KELVIN™ platform (Machine Medicine Technologies, London, UK)	OpenPose	Accuracy 96.51%	NA
	[124]	60	-	Single RGB camera (25 fps)	MobileNetV2 + DUC + DSNT backbone	Overall 5-class accuracy 89.7%	NA
	[125]	128	-	Android tablet (1080p, 30 fps, Google, Mountain View, CA, USA)	YOLO	Accuracy 69.6% (item range: gait 78.1%, face 60.9%); Accuracy 98.8%; MAE 0.32 vs. clinician consensus (>inter-rater MAE 0.65)	NA

Abbreviations: PD = Parkinson’s disease; HC = Healthy controls; NA = not assessed; MDS-UPDRS = Movement Disorder Society Unified Parkinson’s Disease Rating Scale; fps = frames per second; MAE = Mean absolute error; ICC = Intraclass coefficient; SVM = Support Vector Machine; RF = Random Forest; ROI = Region of Interest; AUCROC = Area Under the Curve Receiver Operating Characteristic.

**Table 8:** Diagnostic video analysis algorithms organized by input keypoints.

Ref.	Input/Representation	Model Family	Pose Estimation Software	Task	Performance
[106]	2D keypoints	Feature-based + classical ML	OpenPose	Gait	AUC 0.98
[87]		Detector + features/ML	YOLOv3	Bradykinesia	Accuracy 81.2%
[100]		Feature-based + ML	OpenPose	Gait	AUC 0.91
[107]		Feature-based + ML	OpenPose	Gait	AUC 0.96–0.99
[89]		Feature-based + ML	DeepLabCut	Bradykinesia	AUC 0.968
[92]		Sequence model on skeleton	OpenPose	Gait	AUC 0.91 Accuracy 0.86
[93]		Feature-based + ML	MediaPipe	Bradykinesia	Accuracy 75–86%
[101]		End-to-end CNN/Deep	OpenPose	Gait	Accuracy 99.4%
[102]		Feature-based + ML	OpenPose	Gait	Accuracy 95.8%
[88]		Feature-based + ML	OpenCV Haar face detector + motion-template gradient	Bradykinesia	Accuracy 95%
[108,119]	3D pose	Feature-based + ML	HRNet + HoT-Transformer + GLA-GCN	Gait	Accuracy 93.3%
[98]		Feature-based + ML	Face++ API	Face expressions	F1 0.99 Precision 0.99 Recall 0.99
[99]		End-to-end CNN/Deep	Deep convolutional neural network	Face expressions	AUC 0.71
[110]		End-to-end CNN/Deep	Dense optical	Gait	Accuracy 100%
[94]	Silhouette/Optical-flow/Motion	No ML	EVM	Tremor	OR 2.67 (95% CI 1.39–5.17
[105]		Feature-based + ML	SPIN; SORT tracking; ablation with OpenPose	Gait	Accuracy 81%
[86]	Raw video	End-to-end CNN/Deep	Victor Dibia Handtracking, preprocessing in OpenCV	Bradykinesia	AUC 0.76

Abbreviations: ML = Machine Learning; CNN = convolutional neural network; EVM = Eulerian video magnification; SORT = Simple Online and Realtime Tracking.

**Table 9:** Summary of included studies focusing on therapeutic management classified according to PICO questions per PD patients and controls enrolled, pose estimation software, recording device, performance, and technology used for validation.

Aim	Ref.	Patients	Controls	Device	Pose Estimation	Performance	Technology Comparison
Motor fluctuations	[89]	31	-	Standard clinic video camera	DeepLabCut	ON vs. OFF state classification Accuracy 65%; Sensitivity 65%; Specificity 65%	NA
	[127]	12	12	Wall-mounted RGB video cameras in an instrumented home (640 × 480 @ 30 fps)	OpenPose	− Automatic STS duration vs. clinician-labeled duration Pearson r = 0.419, *p* = 0.042 − Automatic STS speed vs. clinician-labeled duration: Pearson r = −0.780, *p* < 0.001 − Clinical validity: correlations with MDS-UPDRS III—duration ρ = 0.464 (*p* = 0.022); speed ρ = −0.723 (*p* < 0.001) − Group differences: (1) controls vs. PD ON duration *p* = 0.018; speed *p* < 0.001. (2) PD ON vs. OFF duration Z = −1.835 (*p* = 0.034); speed t = 2.211 (*p* = 0.016)	NA
LIDs	[128]	9	-	Consumer video camera (480 × 640 or 540 × 960, 30 fps)	Convolutional Pose Machines (OpenCV 2.4.9)	Best onset AUC 0.822 (UDysRS = 0.826) Best remission AUC 0.958 (UDysRS = 0.802)	NA
	[129]	36	-	Consumer-grade RGB camera (30 fps)	Convolutional Pose Machines (OpenCV 2.4.9)	Pearson *r* = 0.74–0.83	NA
Therapy response	[135]	5	-	2 × FLIR Blackfly USB 3.0 (FLIR Systems, Wilsonville, OR, USA)	DeepLabCut v2.2b6	DLC tracking > 95% (reprojection error 0.016–0.041 px); auto epoch extraction 80%; SVM overall accuracy 85.7%	NA
	[134]	11	12	Camera consumer ≤ 180 fps (Lumix GH5/, Panasonic Corporation, Kadoma, Japan); GoPro Hero7 (GoPro, San Mateo, CA, USA)	MediaPipe	− Glabellar tap reflex PD did not habituate, whereas HC showed habituation by approximately the fourth tap. Levodopa had no effect on the reflex (*p* = 1). − Spontaneous blinks Higher frequency and longer duration in PD; levodopa reduced frequency toward control levels, with a non-significant trend toward shorter duration post-levodopa	NA
	[133]	11		High-frame-rate RGB cameras (≤ 180 fps)	MediaPipe	− Video vs. accelerometer dominant frequency: MAE 0.229 ± 0.174 Hz, RMSE 0.283 Hz, r = 0.98 (*p* = 7.26 × 10^−7^). − Amplitude (video vs. accelerometer): r = 1.00 (*p* = 2.04 × 10^−9^). − Medication effect (video): frequency 2.012 ± 1.385 → * 1.526 ± 1.007 Hz; amplitude 8.167 ± 15.687 → * 4.033 ± 5.671	Accelerometer
	[126]	12	12	Panasonic PV-GS400 CCD camera, 320 × 240, 15 fps (Panasonic Corporation, Kadoma, Japan)	Binary silhouettes → Kernel PCA features	Three-class gait recognition accuracy 80.51% (Non-PD vs. Drug-OFF vs. Drug-ON) using KPCA features	GAITRite^®^ (instrumented walkway)
	[130]	13	-	Tripod-mounted RGB camera, 640 × 480 @ 30 Hz; 6-m walk	Detectron, AlphaPose, OpenPose	Macro-F1 0.22, balanced accuracy 29.5%. Model-predicted MDS-UPDRS-gait scores were lower ON vs. OFF (*p* = 0.017, Cohen’s d = 0.50); ON–OFF change magnitude correlated with clinicians (Kendall’s τ_b = 0.396, *p* = 0.010).	NA
	[132]	31	-	Single-view consumer smartphone camera	MediaPipe	Best AUC 0.79 (LR; combined body + hand), accuracy ≈ 72% (SVM/LR; combined); body-only AUC up to 0.76; hand-only ~0.69	NA
	[131]	48	15	Single 2D camcorder (1080p, 30 fps), GAITRite pressure mat (CIR Systems Inc, Franklin, NJ, USA)	OpenPose	− ICC > 0.90 for step length, velocity, cadence; turning steps—ICC = 0.90 (vs. manual) − ICC = 0.62 for step-length variability Medication ON increased step length and velocity, and reduced turning steps/time; cadence and variability unchanged. Video-derived metrics correlated with FOG-Q, MDS-UPDRS III (total score and gait item), H&Y stage, postural instability (|ρ| ≥ 0.4; *p* < 0.005 for key measures)	GAITRite^®^ (instrumented walkway, CIR Systems Inc, Franklin, New Jersey, USA)

Abbreviations: PD = Parkinson’s disease; HC = Healthy controls; NA = not assessed; fps = frames per second; FOG = freezing of gait; AUC = Area Under the Curve; MDS-UPDRS = Movement Disorder Society Unified Parkinson’s Disease Rating Scale; UDysRS = Unified Dyskinesia Rating Scale; EVM = Eulerian Video Magnification; MAE = Mean absolute error; ICC = Intraclass coefficient; KPCA = Kernel Principal Component Analysis; STS = sit-to-stand; H&Y = Hoehn and Yahr; SVM = Support Vector Machine; LR = Logistic Regression; RMSE = Root Mean Square Error.; LIDs = Levodopa-induced dyskinesia. * = the arrow indicates the medication effect on frequency and amplitude.

## Data Availability

The raw data supporting the conclusions of this article will be made available by the corresponding author upon reasonable request.

## Supplementary Materials

The following supporting information can be downloaded at: https://www.mdpi.com/article/10.3390/s25206373/s1, Figure S1: Graphical representation of mean values of all two authors’ scores of QUADAS-2 tool items for risk of bias and concerns regarding applicability to the review question; Table S1: Quality metrics of included studies according to QUADAS-2 score, with each cell containing assessments from each author.
