# Supplementary material for: AI Video Analysis in Parkinson’s Disease: A Systematic Review of the Most Accurate Computer Vision Tools for Diagnosis, Symptom Monitoring, and Therapy Management
Source: Sensors (Basel). 2025 Oct 15;25(20):6373. doi: 10.3390/s25206373 (PMC12568243; doi:10.3390/s25206373)
Supplement: Supplementary file 1 [file sensors-25-06373-s001.zip › sensors-3862376-Table S1.pdf]

Table S1. Quality metrics of included studies according to QUADAS-2 score with each cell containing assessments from each Author.

Ref.	Au.	Risk of bias				Concerns regarding applicability		
		**PATIENT SELECTION**	**INDEX TEST**	**REFERENCE STANDARD**	**FLOW AND TIMING**	**PATIENT SELECTION**	**INDEX TEST**	**REFERENCE STANDARD**
[1]	1	Unclear	Unclear	Low	Low	Unclear	Low	Low
	2	Low	Low	Low	Low	Low	Low	Low
[2]	1	Low	Low	Unclear	Unclear	Low	Low	Unclear
	2	Low	Unclear	Low	Low	Low	Low	Low
[3]	1	Low	Unclear	Unclear	Unclear	Low	Low	Low
	2	Low	Low	Low	Low	Low	Low	Low
[4]	1	Low	Unclear	Low	Low	Unclear	Low	Low
	2	Low	Unclear	Low	Low	Unclear	Low	Low
[5]	1	Low	Low	Low	Low	Low	Low	Low
	2	Low	Low	Unclear	Unclear	Low	Low	Unclear
[6]	1	Unclear	Low	Low	Low	Low	Low	Low
	2	Low	Unclear	Low	Low	Unclear	Low	Low
[7]	1	Unclear	Low	Low	Low	Low	Low	Unclear
	2	Low	Low	Unclear	Unclear	Low	Low	Low
[8]	1	Low	Low	Low	Low	Unclear	Low	Low
	2	Unclear	Unclear	Low	Low	Low	Low	Low
[9]	1	Low	Low	Low	Unclear	Low	Low	Low
	2	Low	Low	Low	Low	Low	Low	Low
[10]	1	Unclear	Low	Low	Low	Low	Low	Low
	2	Unclear	Unclear	Low	Low	Unclear	Low	Low
[11]	1	Low	Unclear	Low	Low	Low	Low	Low
	2	Unclear	Low	Low	Low	Low	Low	Low
[12]	1	Low	Unclear	Unclear	Unclear	Unclear	Low	Unclear
	2	Low	Unclear	Unclear	Low	Low	Low	Unclear
[13]	1	Low	Low	Low	Low	Low	Low	Low
	2	Low	Low	Low	Low	Low	Low	Low
[14]	1	Unclear	Low	Low	Low	Low	Low	Low
	2	Low	Unclear	Low	Unclear	Unclear	Low	Low
[15]	1	Low	Unclear	Low	Low	Low	Low	Low
	2	Low	Low	Low	Low	Low	Low	Low
[16]	1	Low	Low	Unclear	Low	Unclear	Low	Unclear
	2	Low	Unclear	Low	Low	Low	Low	Low
[17]	1	Low	Low	Low	Unclear	Low	Low	Low
	2	Low	Low	Low	Low	Low	Low	Low
[18]	1	Unclear	Low	Low	Low	Low	Low	Low
	2	Low	Unclear	Unclear	Low	Low	Low	Unclear
[19]	1	Low	Low	Low	Low	Low	Low	Low
	2	Low	Low	Low	Low	Unclear	Low	Low
[20]	1	Unclear	Low	Low	Unclear	Low	Low	Low
	2	Low	Unclear	Low	Unclear	Low	Low	Unclear
[21]	1	Low	Low	Low	Low	Low	Low	Low
	2	Low	Low	Low	Low	Low	Low	Low
[22]	1	Unclear	Low	Unclear	Unclear	Unclear	Low	Unclear
	2	Low	Unclear	Low	Low	Low	Low	Low
[23]	1	Low	Low	Low	Low	Low	Low	Low
	2	Low	Low	Low	Low	Low	Low	Low
[24]	1	Unclear	Low	Unclear	Unclear	Unclear	Low	Low
	2	Low	Unclear	Unclear	Unclear	Low	Low	Unclear
[25]	1	Low	Low	Low	Low	Low	Low	Low
	2	Unclear	Low	Low	Low	Low	Low	Low

Ref.	Au.	Risk of bias				Concerns regarding applicability		
		PATIENT SELECTION	INDEX TEST	REFERENCE STANDARD	FLOW AND TIMING	PATIENT SELECTION	INDEX TEST	REFERENCE STANDARD
[26]	1	Unclear	Low	Low	Low	Low	Low	Low
	2	Low	Unclear	Low	Low	Low	Low	Unclear
[27]	1	Low	Low	Unclear	Unclear	Low	Low	Low
	2	Low	Low	Unclear	Unclear	Low	Low	Low
[28]	1	Low	Low	Low	Low	Low	Low	Low
	2	Unclear	Unclear	Low	Low	Unclear	Low	Unclear
[29]	1	Low	Low	Unclear	Unclear	Low	Low	Low
	2	Low	Low	Low	Low	Low	Low	Low
[30]	1	Low	Low	Low	Low	Low	Low	Unclear
	2	Unclear	Unclear	Unclear	Low	Unclear	Low	Low
[31]	1	Low	Low	Low	Low	Low	Low	Low
	2	Unclear	Low	Unclear	Low	Low	Low	Low
[32]	1	Low	Low	Unclear	Low	Low	Low	Low
	2	Unclear	Unclear	Low	Unclear	Unclear	Low	Unclear
[33]	1	Low	Low	Low	Unclear	Low	Low	Low
	2	Low	Low	Low	Low	Low	Low	Low
[34]	1	Low	Low	Low	Low	Low	Low	Low
	2	Unclear	Unclear	Unclear	Unclear	Low	Low	Low
[35]	1	Low	Low	Low	Low	Low	Low	Low
	2	Low	Low	Low	Low	Low	Low	Low
[36]	1	Unclear	Low	Unclear	Unclear	Low	Low	Unclear
	2	Low	Unclear	Unclear	Unclear	Unclear	Low	Low
[37]	1	Unclear	Low	Low	Low	Low	Low	Low
	2	Low	Low	Low	Low	Low	Low	Low
[38]	1	Low	Low	Unclear	Low	Low	Low	Low
	2	Unclear	Unclear	Low	Low	Unclear	Low	Unclear
[39]	1	Low	Low	Low	Unclear	Low	Low	Low
	2	Unclear	Low	Low	Low	Low	Low	Low
[40]	1	Low	Low	Unclear	Low	Low	Low	Low
	2	Unclear	Unclear	Low	Low	Unclear	Low	Low
[41]	1	Low	Low	Low	Low	Low	Low	Low
	2	Low	Low	Low	Unclear	Low	Low	Low
[42]	1	Low	Low	Unclear	Unclear	Low	Low	Low
	2	Unclear	Unclear	Low	Unclear	Low	Low	Unclear
[43]	1	Low	Low	Low	Low	Low	Low	Low
	2	Unclear	Low	Low	Low	Low	Low	Low
[44]	1	Unclear	Low	Unclear	Unclear	Low	Low	Unclear
	2	Low	Unclear	Unclear	Unclear	Low	Low	Low
[45]	1	Unclear	Low	Low	Low	Low	Low	Low
	2	Unclear	Low	Low	Low	Low	Low	Low

Abbreviations: Au= Author

1. Yang, J., et al., *Deep learning of Parkinson's movement from video, without human-defined measures.* Journal of the Neurological Sciences, 2024: p. 123089.
2. Eguchi, K., et al., *Feasibility of differentiating gait in Parkinson's disease and spinocerebellar degeneration using a pose estimation algorithm in two-dimensional video.* Journal of the Neurological Sciences, 2024. **464**: p. 123158.
3. Heye, K., et al., *Validation of computer vision technology for analyzing bradykinesia in outpatient clinic videos of people with Parkinson's disease.* Journal of the Neurological Sciences, 2024. **466**: p. 123271.

4. Ripic, Z., et al., *Validity of artificial intelligence-based markerless motion capture system for clinical gait analysis: Spatiotemporal results in healthy adults and adults with Parkinson's disease.* Journal of Biomechanics, 2023. **155**: p. 111645.
5. Archila, J., A. Manzanera, and F. Martínez, *A multimodal Parkinson quantification by fusing eye and gait motion patterns, using covariance descriptors, from non-invasive computer vision.* Computer Methods and Programs in Biomedicine, 2022. **215**: p. 106607.
6. Khan, T., et al., *A computer vision framework for finger-tapping evaluation in Parkinson's disease.* Artificial intelligence in medicine, 2014. **60**(1): p. 27-40.
7. Williams, S., et al., *Supervised classification of bradykinesia in Parkinson's disease from smartphone videos.* Artif Intell Med, 2020. **110**: p. 101966.
8. Williams, S., et al., *Seeing the unseen: could Eulerian video magnification aid clinician detection of subclinical Parkinson's tremor?* Journal of Clinical Neuroscience, 2020. **81**: p. 101-104.
9. Li, M.H., et al., *Automated assessment of levodopa-induced dyskinesia: Evaluating the responsiveness of video-based features.* Parkinsonism & related disorders, 2018. **53**: p. 42-45.
10. Liu, P., et al., *Quantitative assessment of gait characteristics in patients with Parkinson's disease using 2D video.* Parkinsonism & Related Disorders, 2022. **101**: p. 49-56.
11. Guarín, D.L., et al., *What the trained eye cannot see: Quantitative kinematics and machine learning detect movement deficits in early-stage Parkinson's disease from videos.* Parkinsonism & Related Disorders, 2024. **127**: p. 107104.
12. Liu, W., et al., *Vision-based estimation of MDS-UPDRS scores for quantifying Parkinson's disease tremor severity.* Medical Image Analysis, 2023. **85**: p. 102754.
13. Chen, S.W., et al., *Quantification and recognition of parkinsonian gait from monocular video imaging using kernel-based principal component analysis.* Biomed Eng Online, 2011. **10**: p. 99.
14. Portilla, J., et al., *A Volumetric Deep Architecture to Discriminate Parkinsonian Patterns from Intermediate Pose Representations.* International Journal of Psychological Research, 2024. **17**(2): p. 84-90.
15. Sabo, A., et al., *Evaluating the ability of a predictive vision-based machine learning model to measure changes in gait in response to medication and DBS within individuals with Parkinson's disease.* BioMedical Engineering OnLine, 2023. **22**(1): p. 120.
16. Li, M.H., et al., *Vision-based assessment of parkinsonism and levodopa-induced dyskinesia with pose estimation.* Journal of neuroengineering and rehabilitation, 2018. **15**: p. 1-13.
17. De Lim, M., et al., *Model-Based Feature Extraction and Classification for Parkinson Disease Screening Using Gait Analysis: Development and Validation Study.* JMIR aging, 2025. **8**(1): p. e65629.
18. Li, T., et al., *Automatic timed up-and-go sub-task segmentation for Parkinson's disease patients using video-based activity classification.* IEEE Transactions on Neural Systems and Rehabilitation Engineering, 2018. **26**(11): p. 2189-2199.
19. Morgan, C., et al., *Automated real-world video analysis of sit-to-stand transitions predicts parkinson's disease severity.* Digital Biomarkers, 2023. **7**(1): p. 92-103.
20. He, R., et al., *A novel multi-level 3D pose estimation framework for gait detection of Parkinson's disease using monocular video.* Frontiers in Bioengineering and Biotechnology, 2024. **12**: p. 1520831.
21. Sato, K., et al., *Quantifying normal and parkinsonian gait features from home movies: Practical application of a deep learning–based 2D pose estimator.* PloS one, 2019. **14**(11): p. e0223549.
22. Shin, J.H., et al., *Automatic measurement of postural abnormalities with a pose estimation algorithm in Parkinson's disease.* Journal of Movement Disorders, 2022. **15**(2): p. 140.
23. Jin, B., et al., *Diagnosing Parkinson disease through facial expression recognition: video analysis.* Journal of medical Internet research, 2020. **22**(7): p. e18697.

24. Abrami, A., et al., *Automated computer vision assessment of hypomimia in Parkinson disease: proof-of-principle pilot study.* Journal of medical Internet research, 2021. **23**(2): p. e21037.
25. Sabo, A., et al., *Concurrent validity of zeno instrumented walkway and video-based gait features in adults with Parkinson's disease.* IEEE Journal of Translational Engineering in Health and Medicine, 2022. **10**: p. 1-11.
26. Williams, S., et al., *Accuracy of smartphone video for contactless measurement of hand tremor frequency.* Movement Disorders Clinical Practice, 2021. **8**(1): p. 69-75.
27. Lu, M., et al. *Vision-based estimation of MDS-UPDRS gait scores for assessing Parkinson's disease motor severity*. in *Medical Image Computing and Computer Assisted Intervention–MICCAI 2020: 23rd International Conference, Lima, Peru, October 4–8, 2020, Proceedings, Part III 23*. 2020. Springer.
28. Guarín, D.L., et al., *Characterizing disease progression in Parkinson's disease from videos of the finger tapping test.* IEEE Transactions on Neural Systems and Rehabilitation Engineering, 2024.
29. Baker, S., et al., *Automatic extraction of upper-limb kinematic activity using deep learning-based markerless tracking during deep brain stimulation implantation for Parkinson's disease: A proof of concept study.* Plos one, 2022. **17**(10): p. e0275490.
30. Kim, J., et al., *Assessment of temporospatial and kinematic gait parameters using human pose estimation in patients with Parkinson's disease: A comparison between near-frontal and lateral views.* PloS one, 2025. **20**(1): p. e0317933.
31. Jansen, T.S., et al., *Video-based analysis of the blink reflex in Parkinson's disease patients.* BioMedical Engineering OnLine, 2024. **23**(1): p. 43.
32. Deng, D., et al., *Interpretable video-based tracking and quantification of parkinsonism clinical motor states.* npj Parkinson's Disease, 2024. **10**(1): p. 122.
33. Rupprechter, S., et al., *A clinically interpretable computer-vision based method for quantifying gait in parkinson's disease.* Sensors, 2021. **21**(16): p. 5437.
34. Chavez, J.M. and W. Tang, *A vision-based system for stage classification of parkinsonian gait using machine learning and synthetic data.* Sensors, 2022. **22**(12): p. 4463.
35. Güney, G., et al., *Video-based hand movement analysis of Parkinson patients before and after medication using high-frame-rate videos and MediaPipe.* Sensors, 2022. **22**(20): p. 7992.
36. Aldegheri, S., et al., *Camera-and viewpoint-agnostic evaluation of axial postural abnormalities in people with Parkinson's disease through augmented human pose estimation.* Sensors, 2023. **23**(6): p. 3193.
37. Simonet, A., et al., *Evaluation of the margin of stability during gait initiation in Young healthy adults, elderly healthy adults and patients with Parkinson's disease: a comparison of force plate and markerless motion capture systems.* Sensors, 2024. **24**(11): p. 3322.
38. Shin, J.H., et al., *Quantitative gait analysis using a pose-estimation algorithm with a single 2D-video of Parkinson's disease patients.* Journal of Parkinson's Disease, 2021. **11**(3): p. 1271-1283.
39. Khan, T., A. Zeeshan, and M. Dougherty, *A novel method for automatic classification of Parkinson gait severity using front-view video analysis.* Technology and Health Care, 2021. **29**(4): p. 643-653.
40. Sarapata, G., et al., *Video-based activity recognition for automated motor assessment of Parkinson's disease.* IEEE Journal of Biomedical and Health Informatics, 2023.
41. Kondo, Y., et al., *Video-based detection of freezing of gait in daily clinical practice in patients with parkinsonism.* IEEE Transactions on Neural Systems and Rehabilitation Engineering, 2024.
42. Guo, Z., et al., *Vision-based finger tapping test in patients with Parkinson's disease via spatial-temporal 3D hand pose estimation.* IEEE Journal of Biomedical and Health Informatics, 2022. **26**(8): p. 3848-3859.

43. Liu, Y., et al., *Vision-based method for automatic quantification of parkinsonian bradykinesia.* IEEE Transactions on Neural Systems and Rehabilitation Engineering, 2019. **27**(10): p. 1952-1961.
44. Xu, J., et al., *Improving reliability of movement assessment in Parkinson's disease using computer vision-based automated severity estimation.* Journal of Parkinson's Disease, 2025: p. 1877718X241312605.
45. Mifsud, J., et al., *Detecting the symptoms of Parkinson's disease with non-standard video.* Journal of neuroengineering and rehabilitation, 2024. **21**(1): p. 72.
