# Supplementary figures and images for: AI Video Analysis in Parkinson’s Disease: A Systematic Review of the Most Accurate Computer Vision Tools for Diagnosis, Symptom Monitoring, and Therapy Management
Source: Sensors (Basel). 2025 Oct 15;25(20):6373. doi: 10.3390/s25206373 (PMC12568243; doi:10.3390/s25206373)

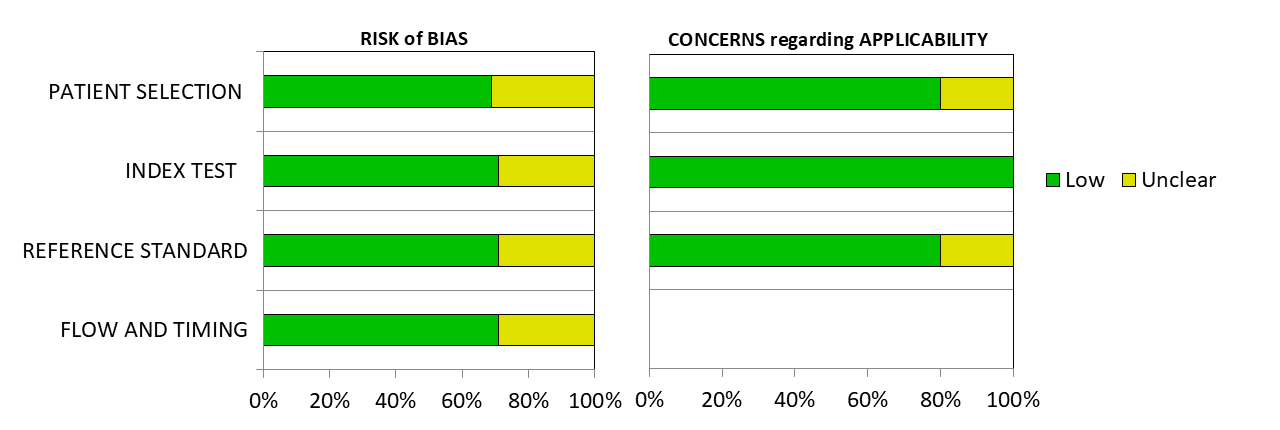

Supplement: Supplementary file 1 [file sensors-25-06373-s001.zip › sensors-3862376-Figure S1.png]
